# OTULIN protects the liver against cell death, inflammation, fibrosis, and cancer

**DOI:** 10.1038/s41418-020-0532-1

**Published:** 2020-03-30

**Authors:** Rune Busk Damgaard, Helen E. Jolin, Michael E. D. Allison, Susan E. Davies, Hannah L. Titheradge, Andrew N. J. McKenzie, David Komander

**Affiliations:** 1 Medical Research Council Laboratory of Molecular Biology, Cambridge Biomedical Campus, Francis Crick Avenue, Cambridge, CB2 0QH UK; 2 Department of Biotechnology and Biomedicine, Technical University of Denmark, Søltofts Plads, 2800 Kgs Lyngby, Denmark; 3 Liver Unit, Department of Medicine, Cambridge Biomedical Research Centre, Cambridge University Hospitals NHS Foundation Trust, Cambridge, CB2 0QQ UK; 4 Department of Histopathology, Cambridge University Hospitals NHS Foundation Trust, Cambridge, CB2 0QQ UK; 5 Birmingham Women’s and Children’s National Health Service Foundation Trust, Mindelsohn Way, Birmingham, B15 2TG UK; 6 Ubiquitin Signalling Division, The Walter and Eliza Hall Institute of Medical Research, Royal Parade, Parkville, Melbourne, VIC 3052 Australia; 7 Department of Medical Biology, The University of Melbourne, Melbourne, VIC 3010 Australia

**Keywords:** Tumour-suppressor proteins, Cell death and immune response, Signal transduction, Chronic inflammation

## Abstract

Methionine-1 (M1)-linked polyubiquitin chains conjugated by the linear ubiquitin chain assembly complex (LUBAC) control NF-κB activation, immune homoeostasis, and prevents tumour necrosis factor (TNF)-induced cell death. The deubiquitinase OTULIN negatively regulates M1-linked polyubiquitin signalling by removing the chains conjugated by LUBAC, and OTULIN deficiency causes OTULIN-related autoinflammatory syndrome (ORAS) in humans. However, the cellular pathways and physiological functions controlled by OTULIN remain poorly understood. Here, we show that OTULIN prevents development of liver disease in mice and humans. In an ORAS patient, OTULIN deficiency caused spontaneous and progressive steatotic liver disease at 10–13 months of age. Similarly, liver-specific deletion of OTULIN in mice leads to neonatally onset steatosis and hepatitis, akin to the ORAS patient. OTULIN deficiency triggers metabolic alterations, apoptosis, and inflammation in the liver. In mice, steatosis progresses to steatohepatitis, fibrosis and pre-malignant tumour formation by 8 weeks of age, and by the age of 7–12 months the phenotype has advanced to malignant hepatocellular carcinoma. Surprisingly, the pathology in OTULIN-deficient livers is independent of TNFR1 signalling. Instead, we find that steatohepatitis in OTULIN-deficient livers is associated with aberrant mTOR activation, and inhibition of mTOR by rapamycin administration significantly reduces the liver pathology. Collectively, our results reveal that OTULIN is critical for maintaining liver homoeostasis and suggest that M1-linked polyubiquitin chains may play a role in regulation of mTOR signalling and metabolism in the liver.

## Introduction

Liver cancer is second most frequent cause of cancer-related deaths worldwide [1]. Nearly all cases of hepatocellular carcinoma (HCC), the most common form of liver cancer, are caused by either chronic liver inflammation (hepatitis) and/or metabolic alterations, which mechanistically are linked to hepatocyte cell death, compensatory regeneration, and excessive mammalian target of rapamycin (mTOR) activation [1–3]. Chronic inflammation and sustained compensatory proliferation induced by hepatocyte damage is pro-tumourigenic and leads to accumulation of mutations and epigenetic changes over time [4, 5]. Pro-inflammatory mediators in the microenvironment support the continuous proliferation and expansion of pre-neoplastic cells, eventually leading to hepatocyte transformation and cancer [6]. Understanding the cellular processes that contribute to the pathogenesis of chronic liver disease resulting in HCC is therefore important to identify new and better therapeutic strategies.

Multiple regulatory mechanisms in inflammation rely on signalling via non-degradative protein ubiquitination [7]. Methionine-1 (M1)-linked ubiquitin (Ub) chains (hereafter referred to as M1-polyUb) are conjugated by the linear Ub chain assembly complex (LUBAC), consisting of the catalytic subunit HOIP and the co-activators HOIL-1 and SHARPIN [8]. M1-polyUb regulates pro-inflammatory nuclear factor-κB (NF-κB) signalling, gene activation, and cell death in response to engagement of tumour necrosis factor (TNF) receptor 1 (TNFR1) and a range of other immune receptors [8, 9]. LUBAC is recruited to the TNFR1 receptor signalling complex where it conjugates M1-polyUb to activate IκB kinase (IKK) and NF-κB [8, 10]. However, without LUBAC and M1-polyUb, TNFR1 signalling is shifted from pro-inflammatory gene activation towards induction of cell death [10, 11], which can occur via caspase-dependent apoptosis or caspase-independent necroptosis [12–17]. Dysregulated TNFR1 and NF-κB signalling have been implicated in the pathogenesis of hepatitis and HCC [2, 18], and several studies have linked regulators of M1-polyUb signalling, including NF-κB essential modulator (NEMO) and HOIP, to the development of liver disease and cancer [19–21].

Ub signalling is antagonised by deubiquitinases (DUBs), which cleave the polyUb signal from substrates to terminate signalling [22]. OTU DUB with linear linkage specificity (OTULIN) and CYLD are the two main DUBs that regulate M1-polyUb signalling [23, 24]. OTULIN exclusively cleaves M1 linkages [25, 26], whereas CYLD cleaves both M1 and K63 linkages [27]. OTULIN binds directly to the LUBAC subunit HOIP [28–30] and regulates LUBAC signalling, autoubiquitination, and stability [25, 31–35]. In humans, homozygous mutations in *OTULIN* cause OTULIN-related autoinflammatory syndrome (ORAS) (also known as otulipenia or autoinflammation, panniculitis, and dermatosis syndrome; OMIM #617099), a life-threatening autoinflammatory disease characterised by fevers, panniculitis, diarrhoea, and arthritis [31, 32, 36, 37]. The primary driver of inflammation in OTULIN-deficient humans and mice is TNF signalling [31, 36], which in myeloid cells leads to LUBAC hyper-signalling and NF-κB activation [31, 32]. In other cell types, e.g. fibroblasts, OTULIN loss leads to LUBAC degradation and TNF-induced cell death [32, 33]. CYLD acts as a tumour suppressor and is mutated in a range of human cancers [38]. However, it remains unknown if OTULIN deficiency also promotes development of cancer or other pathologies.

In this study, we identify OTULIN as critical for preventing liver disease in mice and humans. We demonstrate that OTULIN deficiency causes steatohepatitis, fibrosis, and HCC in mice. Surprisingly, the liver pathology is independent of TNFR1 signalling, but partially dependent on mTOR activity. Consistently, treatment with the mTOR inhibitor rapamycin reduces liver pathology in OTULIN-deficient mice.

## Materials and methods

### Mice

The *Otulin*^del/flox^ and *Otulin*-*Rosa26*-Cre-ERT2 mice, and the generation of bone marrow chimeras were described previously [31]. For chimeras, sex-matched 4–5-month-old *Rosa26-*Cre-ERT2-*Otulin*^+/flox^ (Control^Chim^) or -*Otulin*^del/flox^ (*Otulin*-KO^Chim^) mice were used as recipients. After reconstitution, three doses of tamoxifen (Sigma, St. Louis, MO; 1 mg in sunflower oil with 10% ethanol per dose) were given i.p. to induce OTULIN deletion [31]. *Otulin*^∆hep^ mice with deletion of OTULIN in hepatocytes were generated by breeding *Otulin*^del/flox^ mice with mice expressing Cre from a serum albumin promoter (*Alb*-Cre) [39]. Experimental *Otulin*^∆hep^ mice were either *Otulin*^flox/flox^; *Alb*-Cre^Tg+^ or *Otulin*^del/flox^; *Alb*-Cre^Tg+^. Control mice were *Otulin*^+/flox^; *Alb*-Cre^Tg+^ or occasionally wild type C57BL/6. Mice were matched for age and sex whenever possible. No method of randomisation was applied. All mice were housed under specific pathogen-free conditions. *Tnfr1*^−/−^ mice [40] and the *Alb*-Cre mice were obtained from The Jackson Laboratory, Bar Harbor, ME. All experiments were conducted with the approval of the United Kingdom Home Office and the MRC Centre Ethical Review Committee.

### Human subjects

ORAS Patient IV:3 [31] was evaluated at Birmingham Children’s Hospital, UK. Written informed consent was obtained from the patient and family members. The study was approved by the South Birmingham Research Ethics Committee and performed in accordance with the 1964 Declaration of Helsinki. For further details on patient I:V3, see Damgaard et al. [31].

### Rapamycin treatment of *Otulin*^∆hep^ mice

*Otulin*^∆hep^ and control mice were bred by timed matings. Dams pregnant with pups to be allocated to rapamycin-treated groups received one i.p. injection of rapamycin (1 mg/kg) at E17.5. After birth, pups were allocated to experimental groups based on their genotype and fostered onto pseudopregnant CD-1 mothers. At postnatal day 3 (P3), lactating CD-1 foster mothers received one dose of rapamycin (1 mg/kg) or vehicle s.c. From P8, pups were injected i.p. with rapamycin or vehicle twice weekly until 8 weeks of age. Mice received increasing doses of rapamycin as follows: P3, 20 μg; P11, 25 μg; P15, 30 μg; P18, 35 μg; P22, 135 μg; P25, 180 μg; P29, 240 μg; P32, 240 μg; P36, 300 μg; P39, 300 μg; P43, 330 μg; P46, 330 μg; P50, 330 μg; P53 360 μg; P57, 360  μg; equivalent to 1 mg/kg between P8 and P18 and 3 mg/kg from P22 until the end of the experiment. Mice that met a humane endpoint before the age of 39 days were excluded from analyses. Rapamycin (LC Laboratories, Woburn, MA) was dissolved in 70% ethanol at 20 mg/mL and diluted to 0.2–0.6 mg/mL in sterile PBS containing 0.5% (v/v) Tween-80 (VWR, Lutterworth, UK) and 0.5% (v/v) PEG-400 (Hampton Research, Aliso Viejo, CA) before injection.

### Blood cell counts

Whole blood from terminal bleeds was collected in EDTA-containing Blood Collection Tubes (Greiner GmbH, Kremsmünster, Austria) and analysed on a scil Vet abcPlus^+^ haematological analyser (scil Animal Care Company, Gurnee, IL).

### Histology

Mouse tissue samples were fixed in 10% neutral buffered formalin (Sigma) for 24 h at room temperature. For fresh frozen sections, samples were embedded in OCT Embedding Medium (Thermo Scientific, Waltham, MA). Tissues were sectioned and stained with Haematoxylin and Eosin (H&E), picro sirius red (PSR), periodic acid–schiff (PAS), and Oil Red O at AML Laboratories, Inc., Jacksonville, FL, or Cambridge Stem Cell Institute Histology Core Facility, University of Cambridge, UK. Patient biopsies were processed and H&E stained at Birmingham Children’s Hospital, UK.

### Immunohistochemistry (IHC) and TUNEL assay

All stainings were performed on FFPE sections. For IHC, antigen retrieval was performed in citric acid buffer, pH 6.0, for 15 min at 100 °C. Slides were incubated with primary antibodies (anti-OTULIN, Abcam, or anti-Ki67, Thermo Scientific; see Table S1) at 4 °C overnight and secondary biotinylated antibodies for 30 min at room temperature. Secondary antibodies were labelled using the VECTASTAIN ABC HRP Kit (cat# PK-4001, Vector Laboratories, Burlingame, CA) and detected using the DAB (3,3′-diaminobenzidine) Peroxidase (HRP) Substrate Kit (cat# SK-4100, Vector Laboratories). TUNEL (terminal deoxynucleotidyl transferase dUTP nick end labelling) assays were performed using the ApopTag Peroxidase In Situ Apoptosis Detection kit (cat# S7100, Merck Millipore, Burlington, MA).

### Micrographs and image analysis

Micrographs were taken on an Axioplan microscope (Carl Zeiss) mounted with a Leica DFC310 FX camera using the Leica LAS software. Contrast, brightness, and colour balance were adjusted using Adobe Photoshop. Counting of stained cells, nuclear diameter measurements, and analysis of fibrotic area were performed in the ImageJ or Fiji software. Scale bars represent 200 μm unless otherwise indicated. Image analyses were not blinded.

### Serum and plasma analyses

Serum concentrations of mouse alpha-Fetoprotein (AFP) and mouse insulin were measured using the Mouse AFP Quantikine ELISA Kit (cat# MAFP00; R&D Systems, Minneapolis, MN) and Mouse/Rat Insulin Kit (cat# K152BZC-3; MesoScale Discovery, Rockville, MD), respectively. Serum levels of mouse albumin, bilirubin, glucose, triglycerides, cholesterol, alanine aminotransferase (ALT), and aspartate aminotransferase (AST) were measured on a Dimension EXL Analyser (Siemens Healthcare, Erlangen, Germany) using the DF13, DF167, DF30, DF69A, DF27, DF143, and DF41A cartridges (Siemens Healthcare), respectively. Patient ALT, γ-glutamyl transpeptidase (γGT), and alkaline phosphatase (ALP) levels were measured in plasma using a Vitros 250 or Vitros 750 analyser (Johnson & Johnson Clinical Diagnostics, Rochester, NY).

### Flow cytometry

Analysis of chimerism in Control^Chim^ and *Otulin*-KO^Chim^ mice was performed on splenocytes as previously described [31]. Splenocytes were stained with BrilliantViolet-510-coupled anti-CD45.1 (BioLegend, San Diego, CA) and AlexaFluor-700-coupled anti-CD45.2 (eBioscience, San Diego, CA).

### Purification of endogenous polyUb conjugates

GST-tagged TUBE and M1-SUB were purified from *Escherichia coli* as previously described [32] and endogenous polyUb conjugates were purified from mouse livers as described previously [32, 34, 35]. Briefly, liver tissue was lysed on a TissueLyser II (QIAGEN, Hilden, Germany) in TUBE buffer [32, 34, 35]. GST-tagged TUBE (50 μg/mL) or M1-SUB (100 μg/mL) was added to the lysis buffer immediately before lysis and the lysate incubated with Glutathione Sepharose 4B resin (GE Healthcare, Chicago, IL) for 16–20 h at 4 °C on rotation. Bound material was released by mixing the resin with 1× sample buffer (50 mM Tris pH 6.8, 10% (v/v) glycerol, 100 mM DTT, 2% (w/v) SDS, and 0.01% (w/v) bromophenol blue).

### Immunoblotting

Mouse livers were lysed in RIPA buffer (50 mM Tris pH 7.4, 1% NP-40 (v/v), 0.5% deoxycholate (w/v), 0.1% SDS (w/v), 150 mM NaCl, 2 mM EDTA, and 5 mM MgCl_2_) supplemented with complete protease inhibitor cocktail (Roche, Basel, Switzerland) and PhosSTOP phosphatase inhibitor (Roche) on a TissueLyser II (QIAGEN) as previously described [31]. Samples were resolved on 4–12% Bis-Tris NuPAGE or Novex WedgeWell 4–20% Tris-Glycine gels (Life Technologies, Carlsbad, CA) and transferred to nitrocellulose or PVDF membranes. Membranes were blocked in 5% (w/v) skimmed milk powder dissolved in TBS + 0.1% (v/v) Tween-20 (TBS-T) and incubated with primary antibodies in TBS-T + 3% (w/v) BSA (Sigma). After washing, blots were incubated with HRP-coupled secondary antibodies and visualised using Clarity Western or Clarity Max ECL Substrate (Bio-Rad) on a ChemiDoc MP imager (Bio-Rad). Primary and secondary antibodies are listed in Table S1.

### Quantitative real-time PCR

Total RNA was extracted from mouse liver using the RNeasy Mini Kit (QIAGEN). Liver tissue was lysed in buffer RLT on a TissueLyser II (QIAGEN). Reverse transcription and real-time PCR were performed as previously described [32]. See Table S2 for primer sequences.

### Nuclei isolation and DNA content analysis

Isolation of nuclei from livers of 8-week-old *Otulin*^∆hep^ and control mice and analysis of their DNA content was performed as previously described [41].

### Statistics

Data are presented as individual data points or as means ± SD or SEM as indicated in figure legends. Red bars represent means. Sample number (*n*) represents the number of independent biological samples in each experiment. Sample sizes were estimated from pilot experiments. Data were analysed using the unpaired, two-sided Student’s *t* test of the null hypothesis as indicated. Differences in means were considered statistically significant at *P* < 0.05. Significance levels are: *P < 0.05; **P < 0.01; ***P < 0.001; ****P < 0.0001; n.s., non-significant. Analyses were performed using GraphPad Prism version 7.0b.

## Results

### *Otulin* deletion in non-haematopoietic cells causes acute hepatitis and liver failure

**Fig. 1:**
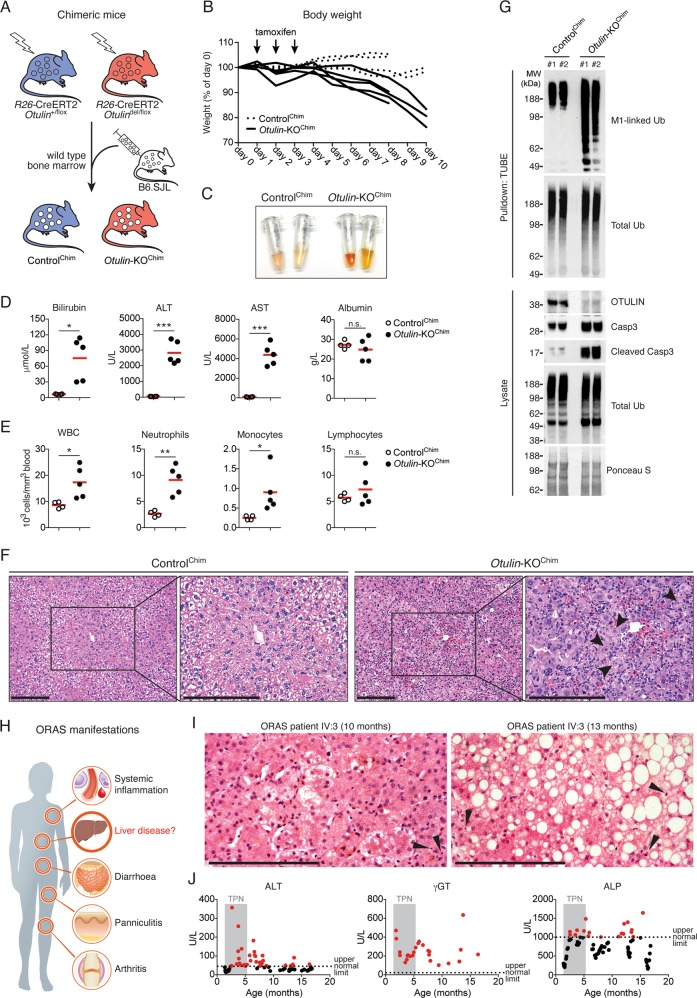
Liver disease in mice and humans deficient in OTULIN. **a** Schematic representation of wild type B6.SJL bone marrow transplantation into γ-irradiated *Rosa26*-Cre-ERT2-*Otulin* (*R26*-Cre-ERT2-*Otulin*) mice. **b** Relative body weight following i.p. administration of tamoxifen (arrows) to Control^Chim^ (*n* = 4) and *Otulin*-KO^Chim^ (*n* = 5) mice. Each line represents one mouse. Data were pooled from two independent experiments. **c** Serum from terminal bleeds of Control^Chim^ and *Otulin*-KO^Chim^ mice at the end of the experiment shown in (**b**). **d, e** Analysis of bilirubin, ALT, AST, and albumin levels in serum (**d**) and blood cell counts (**e**) from terminal bleeds of Control^Chim^ (*n* = 4) and *Otulin*-KO^Chim^ (*n* = 5) mice at the end of the experiment shown in (**b**). Data were pooled from two independent experiments. Data are presented as individual data points, each representing one mouse. Red bars indicate means. Data were analysed using an unpaired, two-sided Student’s *t* test. n.s., non-significant. **f** Micrographs of H&E stained liver sections from Control^Chim^ and *Otulin*-KO^Chim^ mice at the end of the experiments shown in (**b**). Arrowheads indicate cells with nuclear condensation and fragmentation. Micrographs are representative of two mice in each group. **g** Immunoblot analysis of whole-liver lysates and endogenous Ub conjugates purified by TUBE pulldown from livers of two Control^Chim^ and two *Otulin*-KO^Chim^ mice at the end of the experiment shown in (**b**). **h** Schematic representation of the clinical manifestations of ORAS. **i** Micrographs of H&E stained liver sections from an ORAS patient at the age of 10 and 13 months. Arrowheads indicate apoptotic cells. **j** Analysis of ALT, γGT, and ALP levels in plasma from the ORAS patient shown in (**i**). Grey shading indicates period of TPN feeding. Dotted lines indicate upper normal limits of the test. Each data point represents individual measurements, and red data points are above the upper normal limits. See also Fig. S1.

Conditional *Otulin* knockout (KO) mice have revealed cell type-specific phenotypes of OTULIN deficiency in immune cells [31]. However, the role of OTULIN in most non-haematopoietic cell types is unknown. To investigate the function of OTULIN in non-haematopoietic cells, we replaced the bone marrow of *Rosa26*-Cre-ERT2-*Otulin*^flox^ mice [31] with wild type bone marrow to generate chimeric mice that become OTULIN-deficient exclusively in non-haematopoietic cells after tamoxifen administration (*Otulin*-KO^Chim^ mice) (Fig. 1a). *Otulin* deletion by tamoxifen administration resulted in weight loss in *Otulin*-KO^Chim^ mice (Fig. 1b), which was accompanied by highly icteric serum (Fig. 1c) with a ~12-fold increase in the level of the haem metabolite bilirubin (Fig. 1d), indicating potential liver failure in *Otulin*-KO^Chim^ mice. The liver enzymes ALT and AST were also markedly increased in the *Otulin*-KO^Chim^ serum (Fig. 1d), indicating damage to the liver parenchyma, and the number of circulating white blood cells, particularly neutrophils, were elevated in the blood (Fig. 1e). Histological analysis confirmed severe acute hepatitis in the *Otulin*-KO^Chim^ mice with immune cell infiltration and multiple dead or dying hepatocytes with nuclear condensation and fragmentation in the liver (Fig. 1f). In contrast, we observed no obvious pathology in other tissues when compared with Control^Chim^ mice (Fig. S1A).

Immunoblot analysis confirmed efficient deletion of OTULIN in the *Otulin*-KO^Chim^ livers (Figs. 1g and S1B), and tandem Ub-binding entity (TUBE)-mediated enrichment of Ub conjugates showed increased M1-polyUb levels in *Otulin*-KO^Chim^ livers compared with controls. Strikingly, OTULIN deficiency led to marked cleavage and activation of caspase-3 (Fig. 1g), suggesting that the liver pathology in *Otulin*-KO^Chim^ mice could involve apoptosis. Only ~2% of CD45^+^ immune cells present in peripheral tissues in the chimeric mice were of parental origin (Fig. S1C, D), indicating minimal contribution from OTULIN-deficient immune cells to the observed phenotype.

### Steatotic liver disease in an ORAS patient

Intrigued by the severe liver phenotype in *Otulin*-KO^Chim^ mice, we hypothesised that liver disease might be an unrecognised problem in ORAS patients (Fig. 1h). We retrieved and analysed previously unreported liver biopsies and records of liver function tests from an ORAS patient (IV:3) [31]. Liver biopsies from patient IV:3 taken at the age of 10 and 13 months revealed increasing micro- and macrosteatosis, hepatocyte degeneration, and the presence of apoptotic cells, indicating progressive steatotic liver disease (Fig. 1i). Liver function tests from patient IV:3 performed between the age of 6 weeks and her death at 16 months showed ALT, γGT, and ALP levels clearly exceeding the normal range (Fig. 1j), supporting the indication of liver disease in the biopsies. The histopathological changes in the liver and the elevated liver disease markers in plasma persisted long after the intravenous total parenteral nutrition (TPN) was discontinued at the age of ~5 months, showing that the derangements are not side effects of TPN feeding (Fig. 1j).

Collectively, our findings show that OTULIN is required for maintenance of liver homoeostasis in mice and suggest that ORAS patients may develop liver disease in addition to the inflammatory manifestations (Fig. 1h) [31, 36]. We therefore recommend that liver function is monitored closely in known and future cases of ORAS.

### Hepatocyte-specific loss of OTULIN causes spontaneous steatohepatitis, fibrosis, and tumourigenesis

**Fig. 2:**
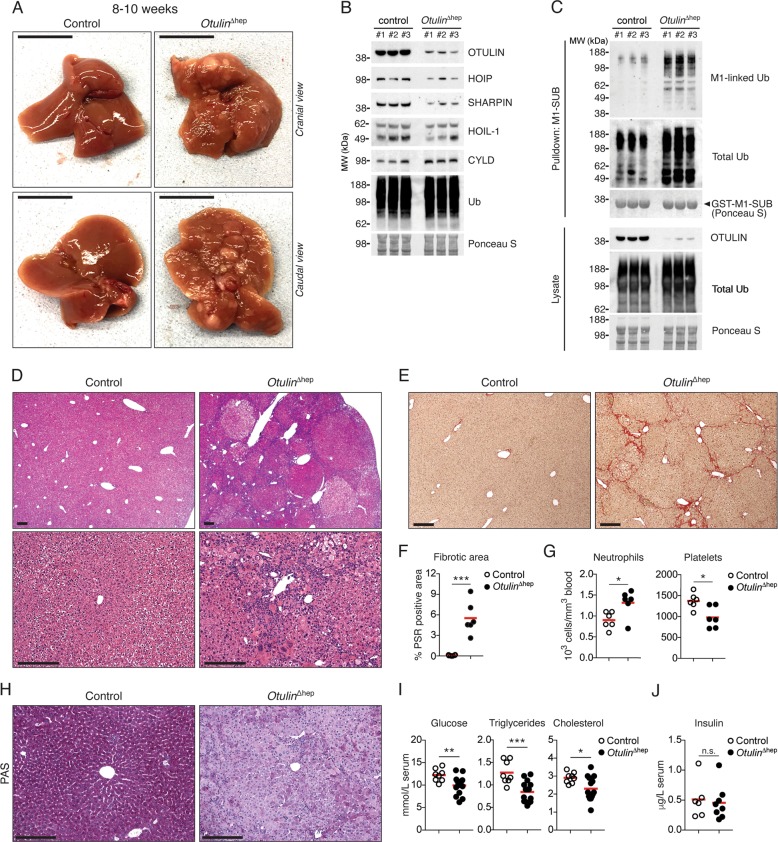
Steatohepatitis, fibrosis, and spontaneous tumour formation in *Otulin*^∆hep^ mice. **a** Representative macroscopic appearance of *Otulin*^∆hep^ and control livers at the age of 8–10 weeks. Scale bars indicate 1 cm. **b** Immunoblot analysis of OTULIN, LUBAC, and CYLD expression in whole-liver lysates from three *Otulin*^∆hep^ and three control mice aged 8–10 weeks. **c** Immunoblot analysis of whole-liver lysates and endogenous Ub conjugates purified by M1-SUB pulldown from livers of three control and three *Otulin*^∆hep^ mice. **d** Micrographs of H&E stained liver sections from *Otulin*^∆hep^ and control mice aged 8–10 weeks. Top panels show pale-staining hepatocyte clones with fat accumulation in *Otulin*^∆hep^ mice. Bottom panels show inflammation, fat accumulation, and variations in nuclear size in *Otulin*^∆hep^ livers. Micrographs are representative of eight mice of each genotype. **e** Micrographs of PSR stained liver sections from *Otulin*^∆hep^ and control mice aged 8–10 weeks show fine bridging porto-portal and porto-central fibrous septa with areas of pericelluar fibrosis in *Otulin*^∆hep^ mice. Micrographs are representative of six mice of each genotype. **f** Quantification of PSR-positive (fibrotic) area in liver sections from *Otulin*^∆hep^ (*n* = 6) and control (*n* = 6) mice aged 8–10 weeks. **g** Neutrophil and platelet counts from terminal bleeds of *Otulin*^∆hep^ (*n* = 6) and control (*n* = 6) mice aged 8–10 weeks. **h** Micrographs of PAS stained liver sections from *Otulin*^∆hep^ and control mice aged 8–10 weeks show pale-staining hepatocytes in *Otulin*^∆hep^ mice due to loss of glycogen. Micrographs are representative of five controls and six *Otulin*^∆hep^ mice. **i**, Analysis of glucose, triglyceride, and cholesterol levels in serum from terminal bleeds of *Otulin*^∆hep^ (*n* = 15) and control (*n* = 8) mice aged 8–10 weeks. **j** Analysis of insulin levels in serum from terminal bleeds of *Otulin*^∆hep^ (*n* = 8) and control (*n* = 6) mice aged 8–10 weeks. **f**, **g**, **i**, **j** Data are presented as individual data points, each representing one mouse. Red bars indicate means. Data were analysed using an unpaired, two-sided Student’s *t* test. n.s., non-significant. See also Fig. S2.

To investigate the role of OTULIN and M1-polyUb signalling in the liver in more detail, we generated mice with hepatocyte-specific deletion of OTULIN (*Otulin*^∆hep^ mice) (Fig. S2A). *Otulin*^∆hep^ mice were born at the expected Mendelian frequency but developed obvious liver pathology (Fig. 2a). OTULIN protein levels were efficiently reduced in whole-liver lysates from these mice (Figs. 2b and S2B). Similar to the *Otulin*-KO^Chim^ mice, OTULIN loss caused a concomitant increase in M1-polyUb in *Otulin*^∆hep^ livers (Figs. 2c and S2C), confirming deregulated M1-polyUb signalling. Residual OTULIN expression in *Otulin*^∆hep^ livers can be attributed to incomplete penetrance of *Alb*-Cre-mediated gene deletion in hepatocytes (Figs. 2b and S2D) as well as to non-parenchymal liver cells that are not targeted by *Alb*-Cre. Expression of the LUBAC components HOIP, HOIL-1, and SHARPIN was reduced, similar to the effect of OTULIN deficiency observed in lymphocytes and fibroblasts [31, 32], while CYLD levels remained unchanged (Fig. 2b).

Dissection of livers from young adult *Otulin*^∆hep^ mice aged 8–10 weeks revealed severe liver disease with the presence of multiple macroscopic lesions and nodules (Fig. 2a). Microscopic examination showed markedly abnormal liver histology in the *Otulin*^∆hep^ mice, including focal steatosis, Mallory–Denk bodies, Kupffer cell hyperplasia, and inflammatory foci (Figs. 2d and S2E, F). These alterations are hallmarks of chronic liver disease and non-alcoholic steatohepatitis (NASH) [42]. Consistent with NASH-like disease, PSR staining showed extensive collagen deposition in the *Otulin*^∆hep^ livers (Fig. 2e, f) with bridging septa and pericellular fibrosis (Fig. S2G), resembling the fibrotic lesions in human NASH and cirrhosis [42]. NASH is a risk factor for HCC development [1]. Further examination of the *Otulin*^∆hep^ livers confirmed that many of the lesions observed macroscopically (Fig. 2a) were in fact dysplastic nodules (Figs. 2d and S2F). Across the parenchyma, we observed prominent variation in size of nuclei (anisokaryosis), large cell change, and clone-like growth (Figs. 2d and S2E, F), which are well-established pre-malignant changes [43]. This liver pathology was fully penetrant in all *Otulin*^∆hep^ mice, and we therefore conclude that OTULIN is intrinsically important in hepatocytes for preventing severe liver disease.

Despite the absence of hepatomegaly (Fig. S2H), *Otulin*^∆hep^ mice exhibited additional indications of disease, including increased neutrophil and decreased platelet counts (Fig. 2g), and an increased proportion of hepatocytes with polyploid nuclei (Fig. S2I–L), similar to findings in cirrhotic and NASH livers [42, 44, 45]. Intriguingly, the glycogen content in *Otulin*^∆hep^ livers was severely reduced. PAS staining, which labels polysaccharides, was homogenous and strong in control livers, whereas OTULIN-deficient livers showed weak staining with only diffuse PAS-positive inclusions (Fig. 2h). Reduced glycogen content was associated with decreased serum concentrations of glucose, triglycerides, and cholesterol (Fig. 2i), despite normal insulin levels (Fig. 2j). These results indicate a disruption in metabolic function that could contribute to development of liver disease in *Otulin*^∆hep^ mice.

### OTULIN deficiency in the liver leads to cell death and inflammation

**Fig. 3:**
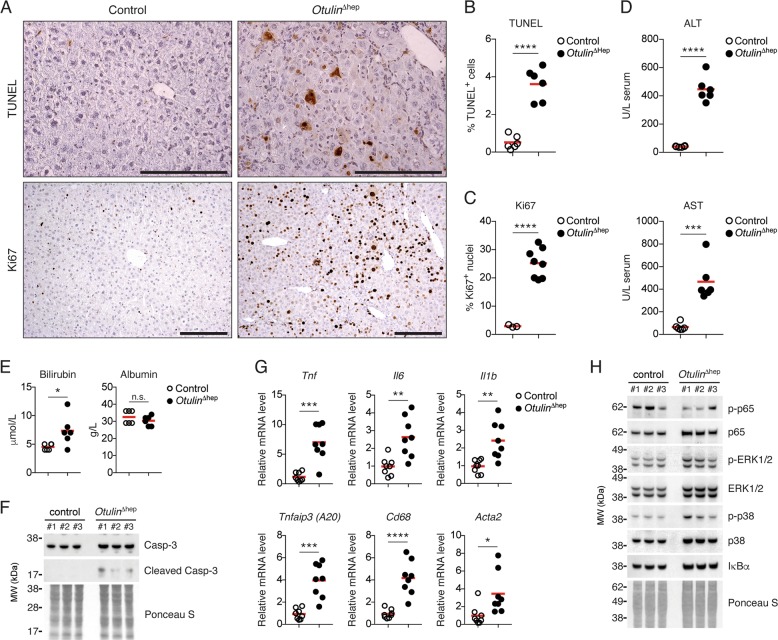
Liver disease in *Otulin*^∆hep^ mice is associated with hepatocyte cell death, proliferation, and inflammation. **a** TUNEL (top panels) and anti-Ki67 (bottom panels) stainings of liver sections from *Otulin*^∆hep^ and control mice aged 8–10 weeks. Data are representative of six mice of each genotype for TUNEL staining and three controls and eight *Otulin*^∆hep^ mice for Ki67. **b**, **c** Quantification of TUNEL- (**b**) and Ki67-positive (**c**) cells in liver from *Otulin*^∆hep^ and control at the age of 8–10 weeks as shown in (**a**). TUNEL (**b**), *Otulin*^∆hep^ (*n* = 6) and control (*n* = 6), and anti-Ki67 (**c**), *Otulin*^∆hep^ (*n* = 8) and control (*n* = 3). **d**, **e** Analysis of ALT and AST (**d**) or bilirubin and albumin (**e**) levels in serum from terminal bleeds of *Otulin*^∆hep^ (*n* = 6) and control (*n* = 6) mice aged 8–10 weeks. **f** Immunoblot analysis of caspase-3 cleavage in whole-liver lysate from livers of three control and three *Otulin*^∆hep^ mice aged 8–10 weeks. **g** Relative mRNA expression of *Tnf*, *Il6*, *Il1b*, *Tnfaip3*, *Cd68*, and *Acta2* in livers from *Otulin*^∆hep^ (*n* = 8) and control (*n* = 8) aged 8–10 weeks measured by quantitative RT-PCR. **h** Immunoblot analysis of NF-κB p65/RelA and MAP kinase activation in whole-liver lysate from livers of three control and three *Otulin*^∆hep^ mice aged 8–10 weeks. **b**–**e**, **g** Data are presented as individual data points, each representing one mouse. Red bars indicate means. Data were analysed using an unpaired, two-sided Student’s *t* test. n.s., non-significant. See also Fig. S3.

Hepatocyte damage and cell death promotes inflammation and NASH development [4]. We investigated if the pathology in *Otulin*^∆hep^ livers was associated with cell death and inflammation. Compared with controls, we observed increased numbers of TUNEL-positive dead cells and Ki67-positive proliferating cells in OTULIN-deficient livers (Fig. 3a–c). Serum from *Otulin*^∆hep^ mice also contained higher levels of ALT, AST, and bilirubin (Fig. 3d, e), consistent with hepatocyte cell death and a moderate reduction in liver function, while albumin levels remained normal (Fig. 3e). Similar to the chimeric mice, immunoblot analysis of liver lysates showed increased caspase-3 activation in *Otulin*^∆hep^ mice compared with controls (Figs. 3f and S3A).

Cell death and proliferation in the *Otulin*^∆hep^ livers was associated with elevated mRNA levels of the pro-inflammatory cytokines TNF, IL-6, and IL-1β as well as the NF-κB and apoptosis regulator A20 (*Tnfaip3*) and the Kupffer cell marker CD68 (Fig. 3g), clearly indicating liver inflammation. Inflammation is a key inducer of collagen-producing myofibroblasts [2]. Consistent with collagen deposition in OTULIN-deficient livers (Fig. 2d), the transcript level of smooth muscle actin (*Acta2*), a myofibroblast marker, was also significantly increased (Fig. 3g), implying myofibroblast expansion. Interestingly, inflammation in the OTULIN-deficient livers was not associated with any appreciable increase in basal NF-κB or MAP kinase activation. Immunoblot analysis showed that phosphorylation of NF-κB p65/RelA and the MAP kinases ERK1/2 and p38 was comparable in *Otulin*^∆hep^ and control livers, as was the expression of the NF-κB inhibitor inhibitor-of-κBα (Figs. 3h and S3B). The absence of increased NF-κB signalling is similar to previous reports from OTULIN-deficient fibroblasts, which are sensitised to induction of apoptosis rather than NF-κB hyper-signalling [32, 33], suggesting that an NF-κB-independent mechanism, e.g. apoptosis or altered metabolism, is responsible for the *Otulin*^∆hep^ pathology.

### Development of HCC in OTULIN-deficient livers

**Fig. 4:**
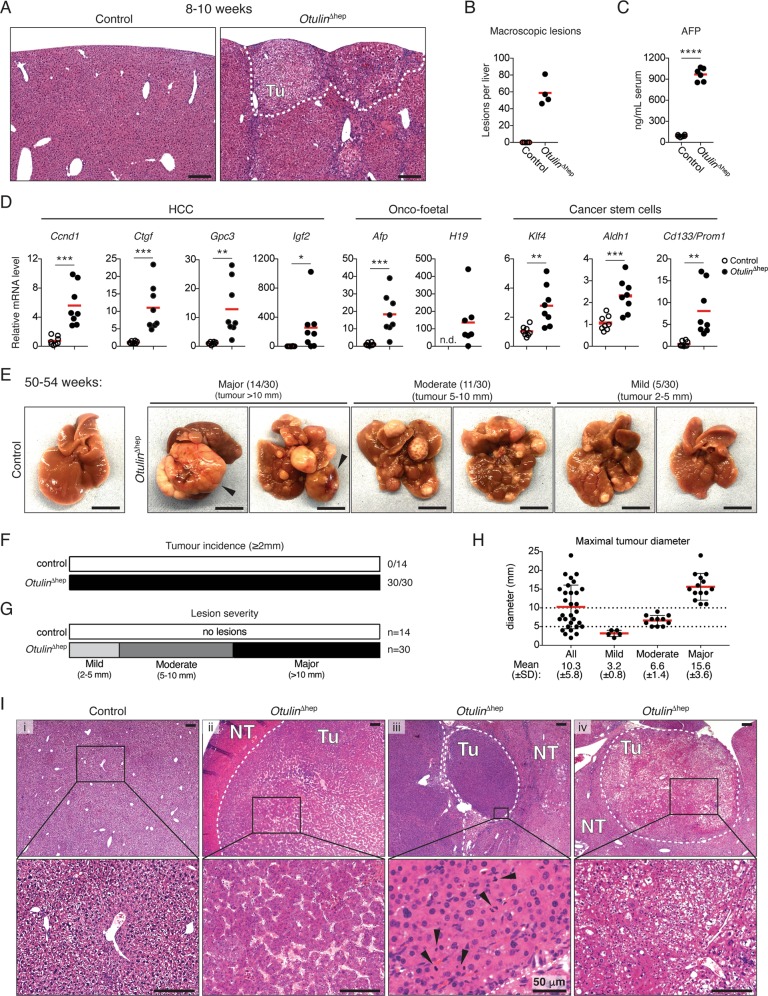
Hepatocellular carcinoma in *Otulin*^∆hep^ mice. **a** Micrographs of H&E stained liver sections from *Otulin*^∆hep^ and control mice aged 8–10 weeks. The dotted line indicates two subcapsular tumours. Micrographs are representative of eight mice of each genotype. Tu, tumour. **b** Quantification of the number of macroscopically discernible lesions (tumours, nodules, and discolourations) in *Otulin*^∆hep^ and control mice aged 8–10 weeks. Data are representative of four mice of each genotype. **c** Analysis of AFP levels in serum from terminal bleeds of *Otulin*^∆hep^ (*n* = 6) and control (*n* = 6) mice aged 8–10 weeks. **d** Relative mRNA expression of the indicated cancer markers in livers from *Otulin*^∆hep^ (*n* = 8) and control (*n* = 8) aged 8–10 weeks measured by quantitative RT-PCR. **e** Representative macroscopic appearance of *Otulin*^∆hep^ and control livers at the age of 50–54 weeks, grouped by severity. Arrowheads indicate highly vascularised tumours. Scale bars indicate 1 cm. **f**, **g** Quantification of the number of mice with the presence of a tumour ≥2 mm in diameter (**f**) or the number of mice in each severity group as indicated (**g**) in *Otulin*^∆hep^ and control mice at the age of 50–54 weeks. **h** Maximal tumour size in *Otulin*^∆hep^ mice, grouped by degree of pathology. Each data point represents the maximal tumour size in one mouse. Red bars indicate means ± SD. **i** Micrographs of H&E stained liver sections from *Otulin*^∆hep^ and control mice aged 50–54 weeks. (i) shows a control liver, (ii) shows HCC with abnormal macrotrabecular pattern, (iii) shows raised nuclear-cytoplasmic ratio, irregular nuclear outlines, and several mitotic figures (arrowheads), and (iv) shows a tumour with a steatohepatic appearance, with also enlarged and irregular nuclei. Micrographs are representative of four controls and 15 *Otulin*^∆hep^ mice. Tu tumour. NT non-tumour. **b**–**d** Data are presented as individual data points, each representing one mouse. Red bars indicate means. Data were analysed using an unpaired, two-sided Student’s *t* test. n.s., non-significant. See also Fig. S4.

Chronic inflammation and NASH predispose to development of HCC [1, 2]. To examine whether the NASH-like pathology in young *Otulin*^∆hep^ mice might lead to cancer, we analysed the OTULIN-deficient livers for signs of neoplasia and HCC. *Otulin*^∆hep^ livers contained multiple pre-malignant tumours (Figs. 2d and 4a) and ~60 macroscopic lesions per liver (Fig. 4b) at 8–10 weeks. The pre-malignant lesions in *Otulin*^∆hep^ livers were accompanied by a dramatic increase in serum levels of the liver cancer marker AFP (Fig. 4c) as well as the expression of many cancer-associated genes, including the HCC markers *Ccnd1*, *Ctgf*, *Gpc3*, and *Igf2*; the onco-foetal markers *Afp* and *H19*; and the cancer stem cell markers *Klf4*, *Aldh1*, and *Cd133/Prom1* (Fig. 4d). This suggested that young *Otulin*^∆hep^ mice were likely to develop HCC.

Indeed, dissection of livers from *Otulin*^∆hep^ mice aged 50–54 weeks revealed the presence of multiple large tumours (Fig. 4e). The tumour incidence (presence of a tumour ≥2 mm in diameter) was 100% in *Otulin*^∆hep^ mice while no lesions were observed in controls (Fig. 4f). While the tumour size, number, and severity varied in *Otulin*^∆hep^ mice, nearly half of them presented with major pathology (tumour > 10 mm in diameter; 14/30) (Fig. 4g). Many mice with major pathology had highly vascularised tumours (Fig. 4e, arrowheads, and S4A). Approximately one third of the *Otulin*^∆hep^ mice developed moderate pathology (tumour 5–10 mm in diameter; 11/30), and only a few mice developed mild pathology (tumour 2–5 mm in diameter; 5/30) (Fig. 4e–h). Microscopic examination uncovered the presence of malignant tumours corresponding to well and moderately differentiated HCC (Fig. 4i) [46]. The analysed tumours were characterised by expansive growth and the absence of portal tracts (Fig. 4i), broad trabecular growth (>4 cells wide) (Fig. 4i, ii), increased eosinophilia (ii and iii) or cell clearance (iv), increased number of mitotic figures (iii, arrowheads), as well as high pleomorphism and atypical nuclei (iv), all indicative of malignant HCC [46]. Occasionally, tumours also showed focal necrosis and cystic degeneration (Fig. S4B), indicating fast-growing and aggressive tumours. Pre-malignant dysplastic nodules with severe anisokaryosis and atypic nuclei were also present [47]. Analysis of *Otulin*^∆hep^ mice aged 32 weeks revealed moderate pathology (Fig. S4C) and the presence of well differentiated tumours (Fig. S4D), occasionally with poor demarcation and the absence of portal tracts, indicating that these are early neoplastic tumours. This indicates that malignancy arises between 32 and 50 weeks of age in *Otulin*^∆hep^ mice.

### Steatohepatitis in *Otulin*^∆hep^ mice is independent of TNFR1 signalling

**Fig. 5:**
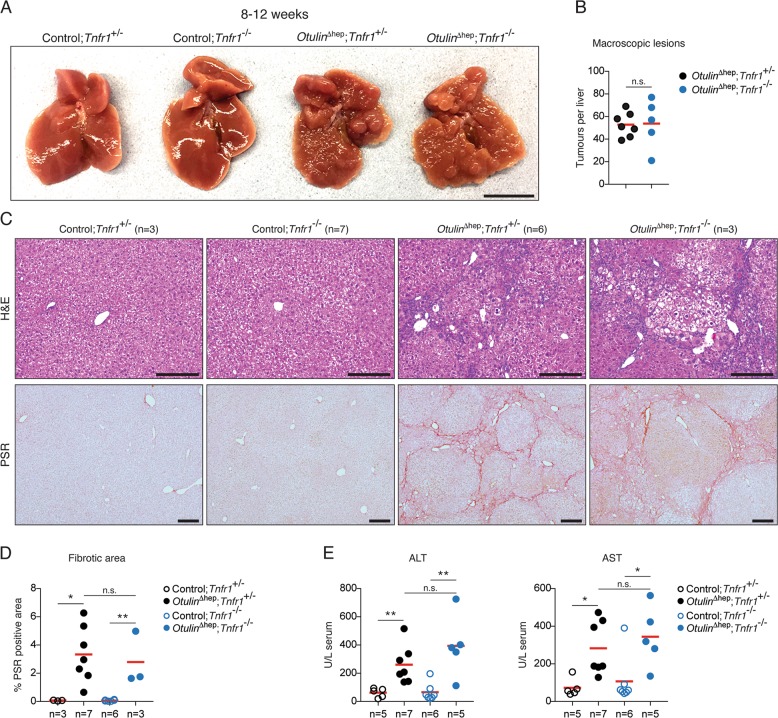
Liver disease in *Otulin*^∆hep^ mice is independent of TNFR1 signalling. **a** Representative macroscopic appearance of livers from *Otulin*^∆hep^ mice, *Otulin*^∆hep^;*Tnfr1*^*−/−*^ mice, and their respective controls at the age of 8–12 weeks. Scale bar indicates 1 cm. **b** Quantification of the number of macroscopically discernible lesions (tumours, nodules, and discolourations) in *Otulin*^∆hep^ (*n* = 7) and *Otulin*^∆hep^;*Tnfr1*^*−/−*^ (*n* = 5) mice aged 8–12 weeks. **c** Micrographs of liver sections from *Otulin*^∆hep^ mice, *Otulin*^∆hep^;*Tnfr1*^*−/−*^ mice, and their respective controls at the age of 8–12 weeks stained with H&E or PSR as indicated. **d** Quantification of PSR-positive (fibrotic) area in liver sections *Otulin*^∆hep^ mice, *Otulin*^∆hep^;*Tnfr1*^*−/−*^ mice, and their respective controls at the age of 8–12 weeks. **e** Analysis of ALT and AST levels in serum from terminal bleeds of *Otulin*^∆hep^ mice, *Otulin*^∆hep^;*Tnfr1*^*−/−*^ mice, and their respective controls at the age of 8–12 weeks. **b**, **d**, **e** Data are presented as individual data points, each representing one mouse. Red bars indicate means. Data were analysed using unpaired, two-sided Student’s *t* tests. n.s., non-significant. See also Fig. S5.

TNF is the primary driver of inflammation in both ORAS patients and ORAS mouse models [31, 32, 36]. Dysregulated TNFR1 signalling also contributes to development of liver disease and cancer [4], and liver-specific deletion of the M1/K63-specific DUB CYLD causes TNFR1-mediated hepatitis and HCC [48]. We therefore investigated if TNFR1 signalling contributed to the liver pathology in *Otulin*^∆hep^ mice. Surprisingly, co-deletion of *Tnfr1* (p55-TNFR1) in *Otulin*^∆hep^ mice did not prevent the development of liver disease (Figs. 5a and S5A). *Otulin*^∆hep^ and *Otulin*^∆hep^;*Tnfr1*^−^^*/−*^ mice aged 8–12 weeks developed indistinguishable pathology (Fig. 5a, b). Microscopic examination revealed virtually identical abnormal histology with dysplastic nodules, large cell change, anisokaryosis, and cytoplasmic inclusions in both *Otulin*^∆hep^ and *Otulin*^∆hep^;*Tnfr1*^*−/−*^ mice (Fig. 5c, top panels, and S5B). The extent and pattern of fibrosis was also unaffected by the deletion of TNFR1 (Fig. 5c, bottom panels, and 5d). Serum levels of ALT and AST, which reflect the degree of cell death in the liver [4], were not significantly reduced in the *Otulin*^∆hep^;*Tnfr1*^*−/−*^ mice either (Fig. 5e), and neither were the cleavage and activation of caspase-3 nor the activation of NF-κB p65/RelA (Fig. S5C). The macroscopic pathology of *Otulin*^∆hep^ and *Otulin*^∆hep^;*Tnfr1*^*−/−*^ mice remained indistinguishable until at least the age of 20–25 weeks (Fig. S5D). We therefore conclude that the cellular aberrations leading to liver disease in *Otulin*^∆hep^ mice are independent of TNFR1 signalling and thus distinct from the pathology in CYLD-deficient livers [48].

### Prominent neonatal steatosis and aberrant mTOR activation in *Otulin*^∆hep^ mice

**Fig. 6:**
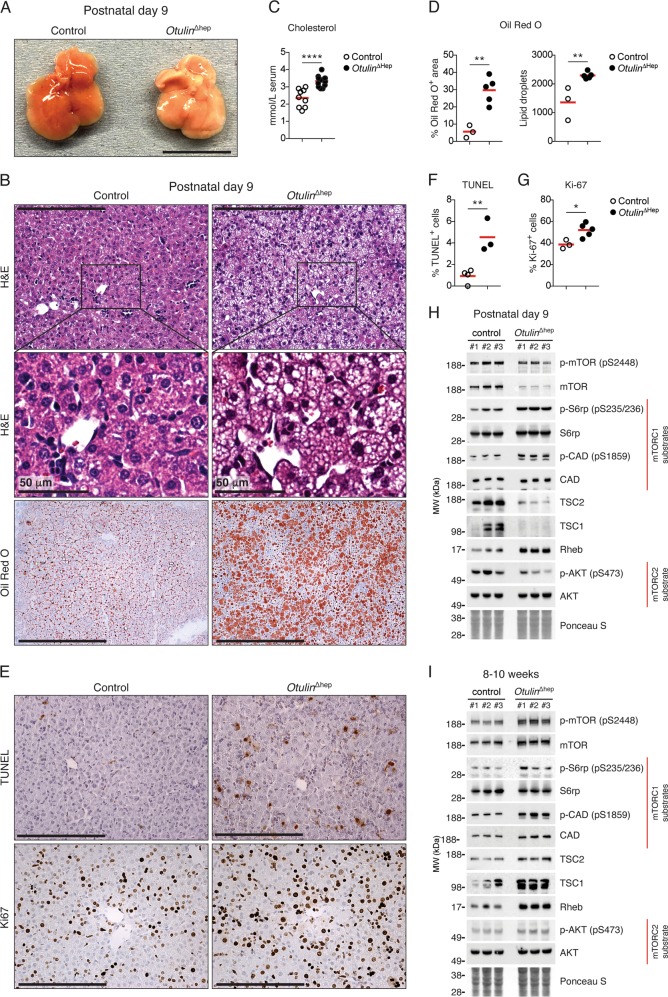
Steatosis and aberrant mTOR activation in neonatal *Otulin*^∆hep^ mice. **a** Representative macroscopic appearance *Otulin*^∆hep^ and control livers at the age of 9 days. Scale bar indicates 1 cm. **b** Micrographs of liver sections from *Otulin*^∆hep^ and control mice at the age of 9 days stained with H&E and Oil Red O as indicated. H&E staining shows pale hepatocytes with varying sized vacuoles in *Otulin*^∆hep^ mice, which is confirmed as fat by Oil Red O staining. Micrographs are representative of seven controls and six *Otulin*^∆hep^ mice for H&E, and three controls and five *Otulin*^∆hep^ mice for Oil Red O. **c** Analysis of cholesterol levels in serum from terminal bleeds of *Otulin*^∆hep^ (*n* = 9) and control (*n* = 6) mice at the age of 9 days. **d** Quantification of Oil Red O-positive area (left) and number of lipid droplets (right) in liver sections *Otulin*^∆hep^ (*n* = 5) and control (*n* = 3) at the age of 9 days as shown in (**b**). **e** TUNEL (top panels) and anti-Ki67 (bottom panels) stainings of liver sections from *Otulin*^∆hep^ and control mice aged 9 days. Data are representative of four control and three *Otulin*^∆hep^ mice for TUNEL, and three controls and five *Otulin*^∆hep^ mice for Ki67. **f**, **g** Quantification of TUNEL- (**f**) and Ki67-positive (**g**) cells in liver from *Otulin*^∆hep^ and control at the age of 9 days as shown in (**e**). TUNEL (**f**), *Otulin*^∆hep^ (*n* = 3) and control (*n* = 4), and anti-Ki67 (**g**), *Otulin*^∆hep^ (*n* = 5) and control (*n* = 3). **h**, **i** Immunoblot analysis of mTOR pathway components and activation in whole-liver lysate from three *Otulin*^∆hep^ mice and three controls aged 9 days (**h**) and 8–10 weeks (**i**). **c**, **d**, **f**, **g** Data are presented as individual data points, each representing one mouse. Red bars indicate means. Data were analysed using an unpaired, two-sided Student’s *t* test. n.s., non-significant. See also Fig. S6.

As the phenotype in young adult *Otulin*^∆hep^ mice was independent of TNFR1 signalling, we examined livers from younger *Otulin*^∆hep^ mice to define the onset of the phenotype. Analysis of neonatal *Otulin*^∆hep^ and control livers at postnatal day (P) 3 and P9 showed that OTULIN ablation was efficient at this age and that HOIP expression was reduced (Fig. S6A–D), similar to our observations at 8–10 weeks. Strikingly, neonatal *Otulin*^∆hep^ mice displayed noticeable steatosis at P3 and P9 (Figs. 6a, b and S6E), akin to the liver disease in the infant ORAS patient (Fig. 1i). The neonatal *Otulin*^∆hep^ livers were pale and oily, particularly at P9 (Fig. 6a). The cholesterol level in serum was also increased at P9 (Fig. 6c), while triglyceride and glucose levels were comparable in *Otulin*^∆hep^ and control mice (Fig. S6F). Histopathological examination indicated progressive lipid accumulation, mainly microsteatosis, between P3 and P9 in *Otulin*^∆hep^ mice (Figs. 6b, top and centre panels, and S6E), and lipid-specific Oil Red O staining confirmed prominent steatosis in the P9 livers (Fig. 6b, bottom panels, and 6d).

Immunohistochemical analysis showed an increase in TUNEL-positive cells in the *Otulin*^∆hep^ livers at P9 (Fig. 6e, top panels, and 6f), comparable with the increase observed at 8–10 weeks. Both at P3 and P9, caspase-3 cleavage was also increased (Figs. S6A, C). The number of Ki67-positive proliferating cells was also elevated in the P9 *Otulin*^∆hep^ livers, although only marginally (Fig. 6e, bottom panels, and 6g), likely due to the fact that the liver at this age is a highly proliferative organ already. In contrast, we did not detect any signs of collagen deposition at either P3 or P9 in these mice (Fig. S6G, H).

The kinase mTOR is a master regulator of cellular metabolism and growth [3], and increased mTOR activity promotes liver cancer development in mice [49, 50]. In models of mTOR-driven carcinogenesis, metabolic alterations accompany hepatocyte damage and proliferation [49, 50], akin to our observations in *Otulin*^∆hep^ mice. We therefore investigated if mTOR signalling was altered in *Otulin*^∆hep^ livers. Intriguingly, we observed aberrant mTOR activity in P9 *Otulin*^∆hep^ livers compared with controls (Fig. 6h and S6D). When compared with the overall lower expression of total mTOR protein in P9 *Otulin*^∆hep^ liver lysates, the relative phosphorylation of the activating Ser2448 in mTOR was increased compared with controls (Fig. 6h). This correlated with increased phosphorylation of the mTOR complex 1 (mTORC1)-dependent substrates S6 ribosomal protein (S6rp) and carbamoyl-phosphate synthetase, aspartate transcarbamylase, and dihydroorotase (CAD) (Fig. 6h). S6rp phosphorylation was also increased in P3 livers (Fig. S6H). Phosphorylation of mTOR and its substrates correlated with reduced levels of the TSC complex (consisting of TSC1 and TSC2), a negative regulator of mTOR, and increased expression of the mTOR activator Rheb in P9 *Otulin*^∆hep^ livers (Figs. 6h and S6I). At 8–10 weeks, OTULIN-deficient livers showed more normal, but still slightly increased, mTORC1 activation (Figs. 6i and S6J). The expression of the TSC complex was comparable with controls, but Rheb expression was still increased (Fig. 6i). We observed no apparent increase in phosphorylation of the mTORC2 substrate Akt (Ser473) (Fig. 6h, i). These findings indicate that OTULIN deficiency leads to aberrant mTORC1 activation.

### mTOR inhibition reduces liver disease in *Otulin*^∆hep^ mice

In humans, mTOR activity is upregulated in 40–50% of HCC cases and is associated with poor prognosis [51]. In mice, increased mTOR activity leads to HCC development, which can be counter acted by treatment with mTOR inhibitors [49, 50]. To examine if OTULIN deficiency led to mTOR-driven liver disease, we tested if inhibition of mTOR could reduce the pathology in the *Otulin*^∆hep^ mice. As aberrant mTOR activation is evident already at P3, we treated *Otulin*^∆hep^ mice with rapamycin from birth until the age of 8 weeks. Rapamycin treatment was not well tolerated in *Otulin*^∆hep^ mice. Treated *Otulin*^∆hep^ mice displayed reduced weight gain when compared with vehicle-treated mice and even rapamycin-treated controls (Fig. S7A), demonstrating a pharmacogenetic interaction between OTULIN deficiency and mTOR inhibition. The condition of the rapamycin-treated *Otulin*^∆hep^ mice meant that for many mice the experiment had to be stopped at 6 weeks of age (Fig. S7A).

**Fig. 7:**
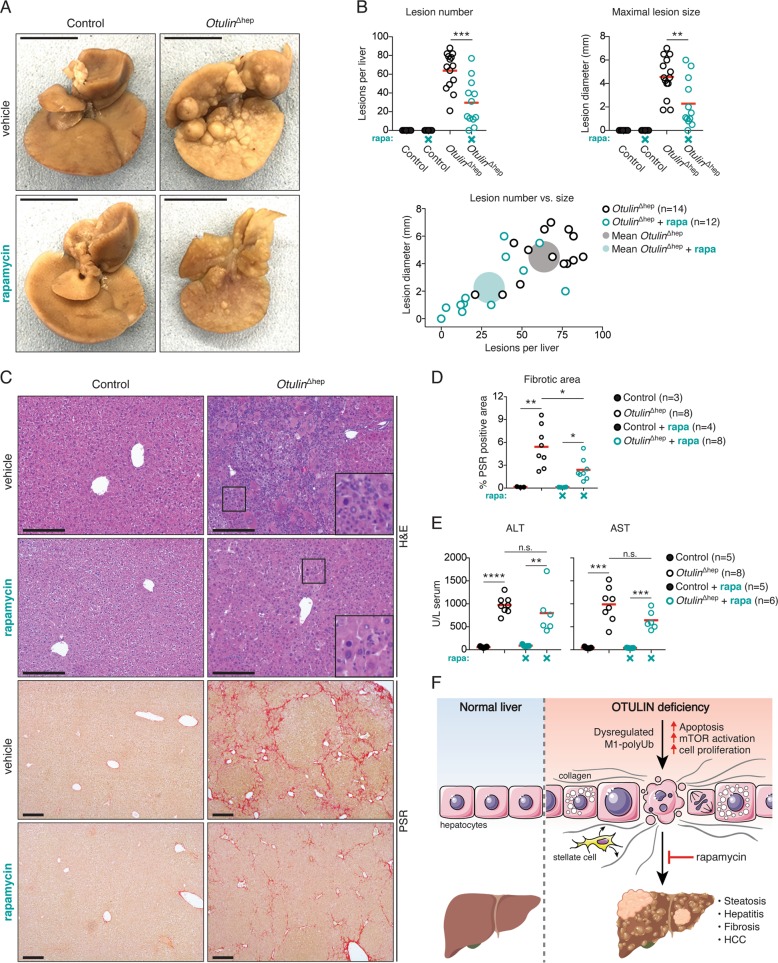
The mTOR inhibitor rapamycin reduces liver pathology in *Otulin*^∆hep^ mice. **a** Representative macroscopic appearance of formalin-fixed livers from *Otulin*^∆hep^ and control mice at the age of 6 weeks treated with rapamycin or vehicle as indicated. Scale bars indicate 1 cm. **b** Quantification of, and correlation between, the number and maximal size of macroscopically discernible lesions (tumours, nodules, and discolourations) in *Otulin*^∆hep^ and control mice aged 6–8 weeks treated with rapamycin (rapa) or vehicle as indicated. Data were pooled from two independent experiments. Opaque circles indicate the mean lesion number and mean maximal lesion size of the two groups. **c** Micrographs of liver sections from *Otulin*^∆hep^ and control mice at the age of 6 weeks treated with rapamycin or vehicle and stained with H&E and PSR as indicated. Data are representative of three vehicle-treated controls, eight vehicle-treated *Otulin*^∆hep^ mice, four rapamycin-treated controls, and eight rapamycin-treated *Otulin*^∆hep^ mice. Inserts show cellular changes at high magnification. **d** Quantification of PSR-positive (fibrotic) area in liver sections from *Otulin*^∆hep^ and control mice at the age of 6 weeks treated with rapamycin (rapa) or vehicle as indicated. **e** Analysis of ALT and AST levels in serum from terminal bleeds from control and *Otulin*^∆hep^ mice at the age of 6–8 weeks treated with vehicle or rapamycin (rapa) as indicated. Data were pooled from two independent experiments. **f** Model of the cellular and phenotypic changes in OTULIN-deficient livers. **b**, **d**, **e** Data are presented as individual data points, each representing one mouse. Red bars indicate means. Data were analysed using unpaired, two-sided Student’s *t* tests. n.s., non-significant. See also Fig. S7.

Remarkably, despite early termination of the experiment, rapamycin treatment reduced the pathology in *Otulin*^∆hep^ livers compared with vehicle-treated *Otulin*^∆hep^ mice of the same age (Fig. 7a). Rapamycin treatment reduced both the number and size of macroscopic lesions in the livers, but it did not completely prevent liver disease (Fig. 7a, b). The livers from the rapamycin-treated *Otulin*^∆hep^ mice appeared smaller than vehicle-treated *Otulin*^∆hep^ mice or rapamycin-treated controls (Fig. 7a), but relative to body weight they were not different from vehicle-treated *Otulin*^∆hep^ livers (Fig. S7B). Microscopically, rapamycin reduced the histological abnormalities and the number of dysplastic foci and nodular growths in the *Otulin*^∆hep^ livers (Figs. 7c and S7C). Hepatocyte dysplasia and the inflammatory cells in the parenchyma were decreased in the rapamycin-treated *Otulin*^∆hep^ livers, although some of the cellular changes, including atypical nuclei and hepatocyte hypertrophy, persisted (Fig. 7c, inserts). In addition, rapamycin treatment significantly reduced fibrosis in *Otulin*^∆hep^ mice (Fig. 7c, d). Importantly, ALT and AST levels in serum were not significantly reduced by mTOR inhibition (Fig. 7e), suggesting that apoptosis in *Otulin*^∆hep^ livers is independent of mTOR. Our findings demonstrate that mTOR activity promotes fibrosis and liver disease in *Otulin*^∆hep^ mice, but also that mTOR inhibition by rapamycin is insufficient to completely prevent liver pathology in these mice.

## Discussion

We provide evidence that OTULIN is a crucial in vivo regulator of liver homoeostasis in mice and humans, identify mTOR signalling as a surprising driver of liver disease in OTULIN-deficient mice, and show that mTOR inhibition with rapamycin can improve liver pathology caused by OTULIN deficiency. In humans, OTULIN deficiency causes a severe autoinflammatory syndrome, ORAS [31, 32, 36, 37], and genetic ablation of OTULIN in immune cells in mice replicate many inflammatory hallmarks of ORAS [31]. Our discovery that OTULIN deficiency also causes severe liver disease in humans and mice expands the range of pathologies associated with OTULIN and highlights the critical importance of proper regulation of M1-polyUb signalling.

*Otulin*^∆hep^ livers exhibit early-onset progressive liver disease. Within days of birth, *Otulin*^∆hep^ mice develop steatosis. As the *Alb*-Cre transgene is expressed in the late foetal stages [52], the neonatal steatosis in *Otulin*^∆hep^ mice may result from signalling responses or other priming events onset before birth. It will be important to delineate these foetal events and their contribution to the phenotype in future studies.

By the age of 8 weeks, the neonatal steatosis in *Otulin*^∆hep^ mice has developed into steatohepatitis, fibrosis, and pre-malignant tumours, and HCC by the age of 7–12 months. This pattern of disease progression—from steatosis to steatohepatitis, fibrosis, and HCC—is remarkably similar to the advancement of liver disease in human NASH patients [1, 42]. NASH-like steatohepatitis and HCC also develops in mice with liver-specific deletion of the M1-polyUb DUB CYLD [48]. In these mice, the apoptosis and compensatory regeneration that drives the pathogenesis is completely dependent on TNFR1. Surprisingly, unlike CYLD-deficient livers, TNFR1 signalling is dispensable for steatohepatitis and pre-malignant tumour development in *Otulin*^∆hep^ livers, clearly indicating that the liver diseases caused by deficiency in the two main M1-polyUb-regulating DUBs, OTULIN, and CYLD, have distinct pathogeneses. In contrast to OTULIN, which is strictly M1 linkage-specific [25, 26], CYLD cleaves both M1 and K63 linkages [27]. The apparent difference in steatohepatitis pathogenesis caused by deficiency in these two DUBs could therefore arise from combined dysregulation of M1- and K63-polyUb signalling in the CYLD-deficient livers but exclusively M1-polyUb dysregulation in OTULIN-deficient livers. In addition, dysregulation of LUBAC complexes could contribute to the observed differences [8]. OTULIN and CYLD form mutually exclusive complexes with LUBAC [53]. In the absence of OTULIN, only CYLD-SPATA2-LUBAC complexes can form [54–57], and conversely, without CYLD, only OTULIN-LUBAC complexes can assemble [28–30]. Dysregulation of the LUBAC-independent OTULIN-SNX27 complex could also be involved [58].

Our examination of neonatal *Otulin*^∆hep^ livers revealed an unexpected phenotype of steatosis and aberrant mTOR signalling. Remarkably, mTOR inhibition by rapamycin administration reduces liver pathology in *Otulin*^∆hep^ mice. To our knowledge, this is the first report of a link between M1-polyUb and mTOR. Both degradative and non-degradative ubiquitination can regulate mTOR pathways [59], but no direct link to M1-polyUb has been reported. OTULIN deficiency appears to cause changes in the expression of mTOR regulators, such as the TSC complex and Rheb, in the liver, likely leading to aberrant mTOR activation. However, the molecular nature of this dysregulation, and whether it is a direct effect or part of a secondary regenerative response, is unclear. Intriguingly, mTOR signalling can regulate inflammatory processes and apoptosis [60], and a link between OTULIN, M1-polyUb, and mTOR could potentially connect the metabolic alterations and inflammation observed in *Otulin*^∆hep^ mice. However, more mechanistic studies are needed to elucidate any molecular link between M1-polyUb and mTOR.

In summary, we demonstrate that OTULIN prevents cell death, inflammation, and metabolic derangements in the liver and can act as a tumour suppressor in mice (Fig. 7f). This highlights how delicately balanced M1-polyUb signalling must be to prevent disease. Moreover, the pathology in OTULIN-deficient livers is partially dependent on mTOR activity and can be ameliorated by rapamycin treatment. This suggests a role for OTULIN in mTOR regulation and implicates M1-polyUb in cellular signalling processes beyond control of NF-κB and TNF-mediated cell death.

## Supplementary information

S1

S2

S3

S4

S5

S6

S7

Supplemental Material
