# Supplementary figures and images for: OTULIN protects the liver against cell death, inflammation, fibrosis, and cancer
Source: Cell Death Differ. 2020 Mar 30;27(5):1457–74. doi: 10.1038/s41418-020-0532-1 (PMC7206033; doi:10.1038/s41418-020-0532-1)

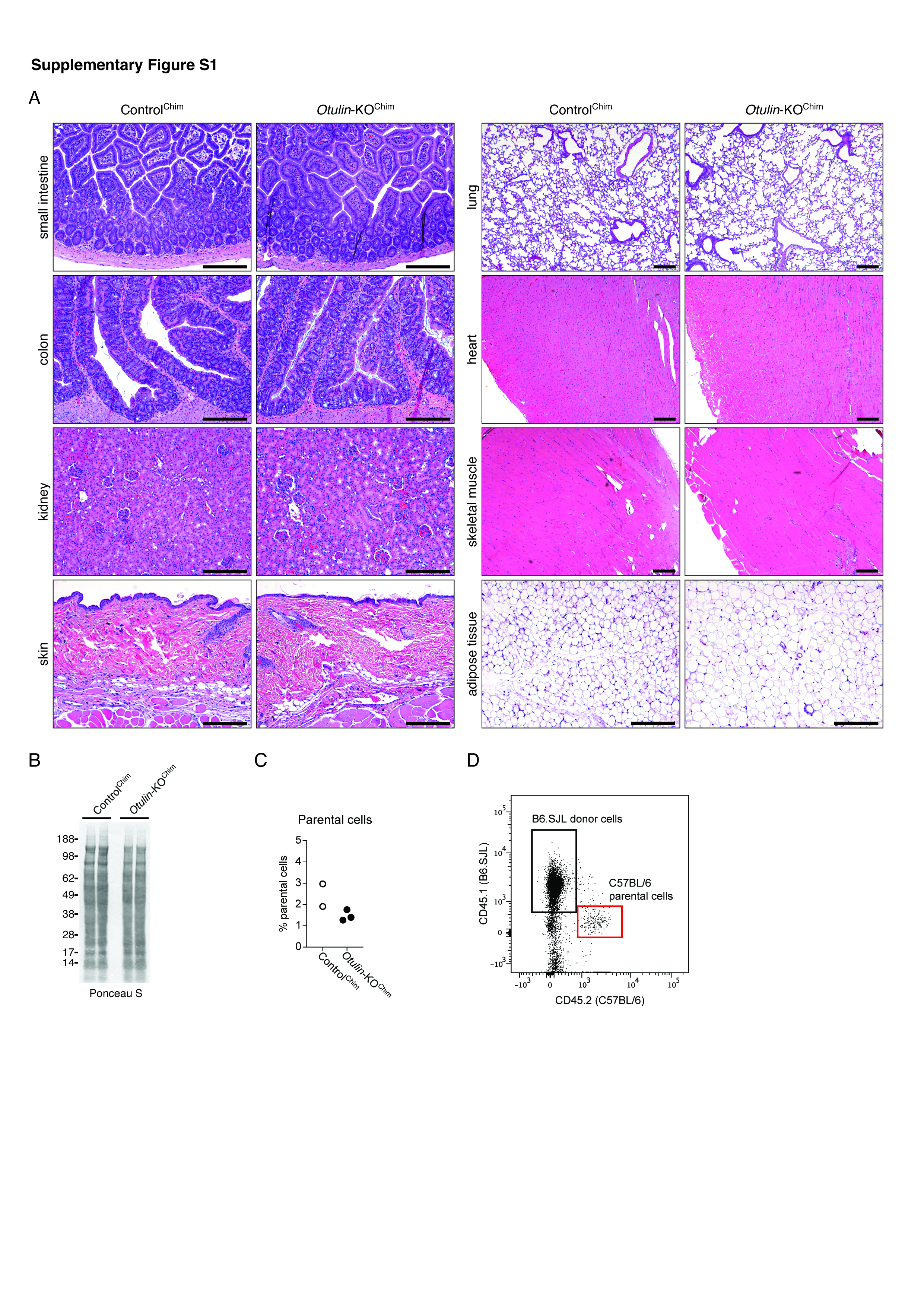

Supplement: Supplementary file 1 — S1 [file 41418_2020_532_MOESM1_ESM.tif]

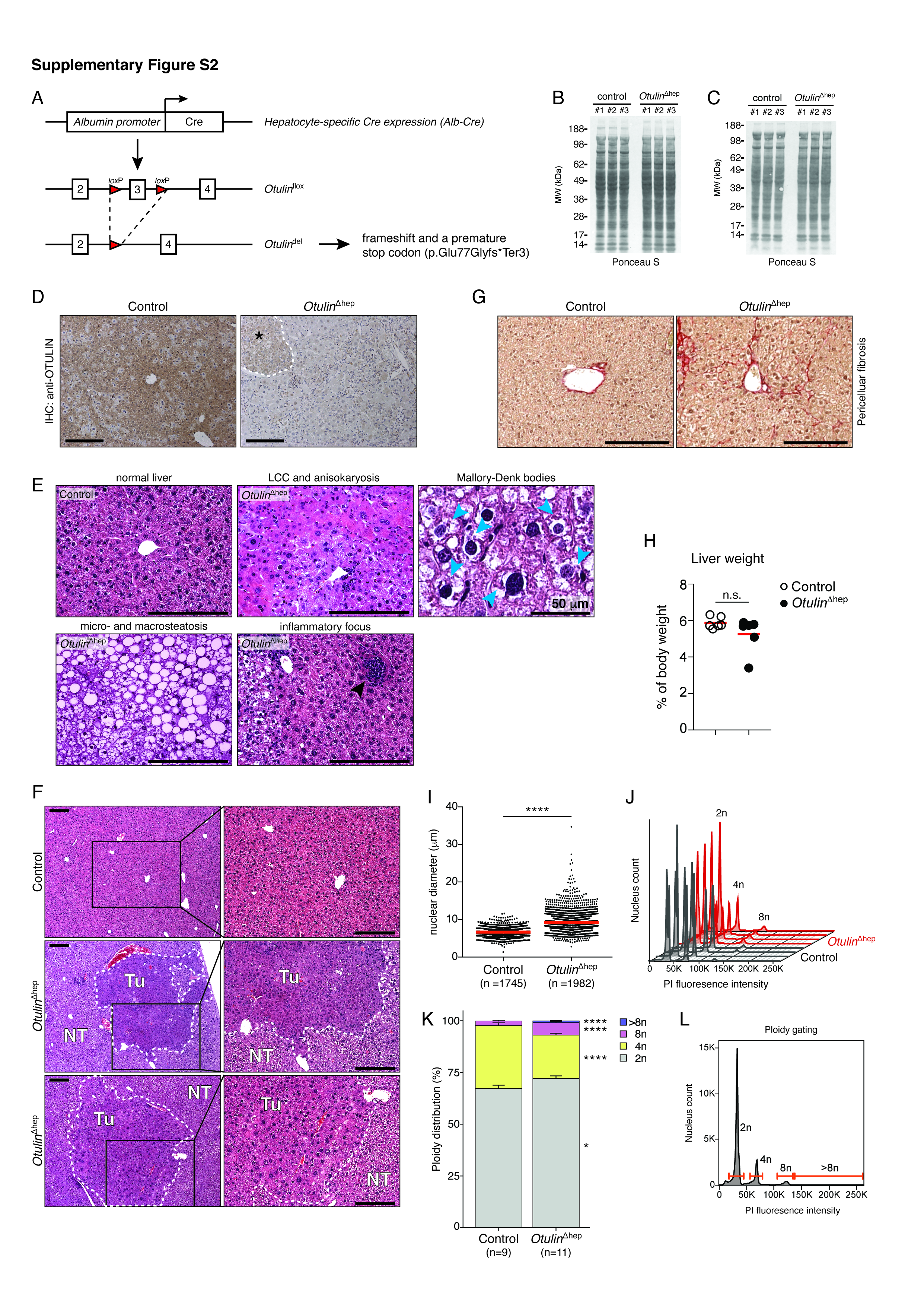

Supplement: Supplementary file 2 — S2 [file 41418_2020_532_MOESM2_ESM.tif]

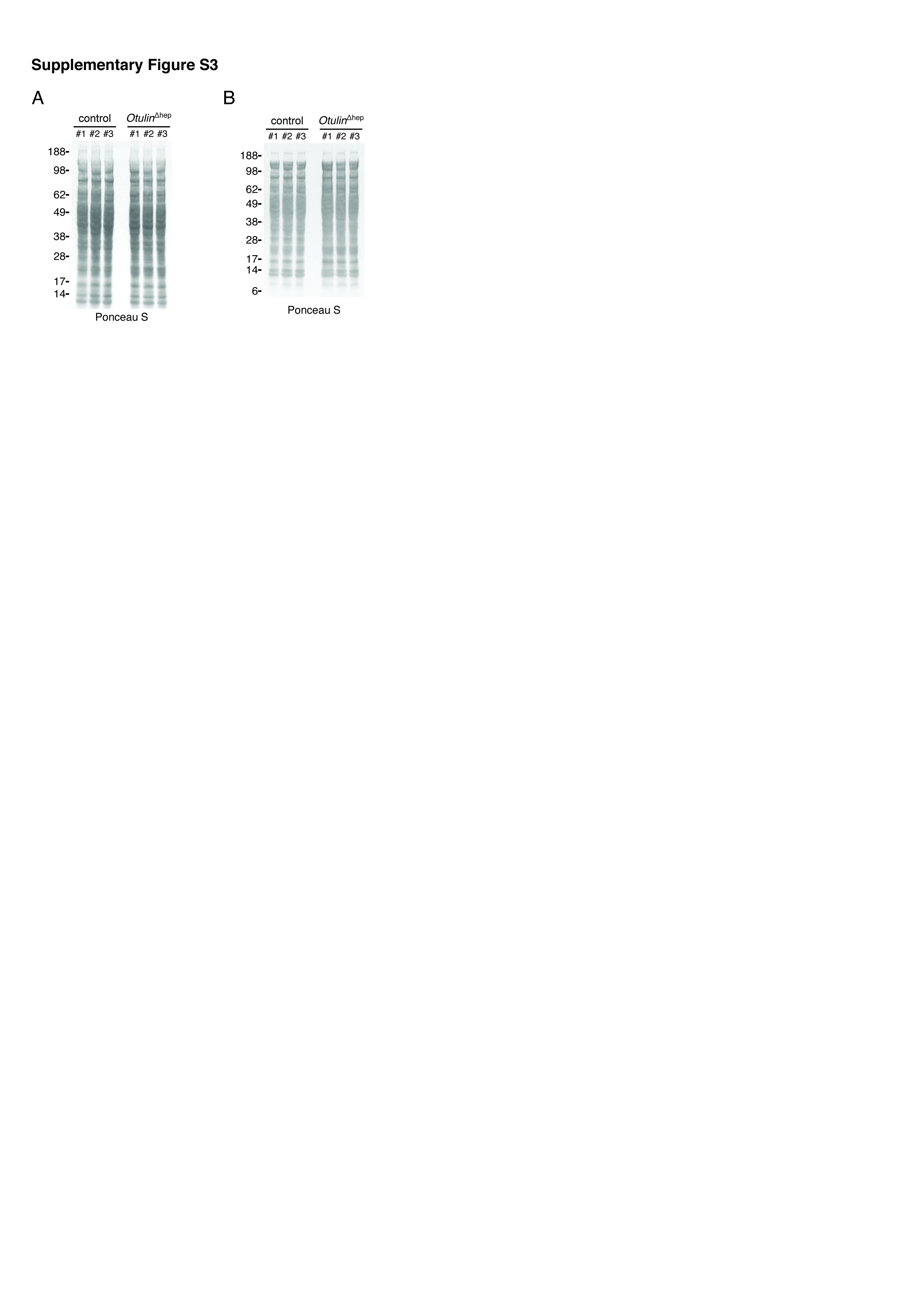

Supplement: Supplementary file 3 — S3 [file 41418_2020_532_MOESM3_ESM.tif]

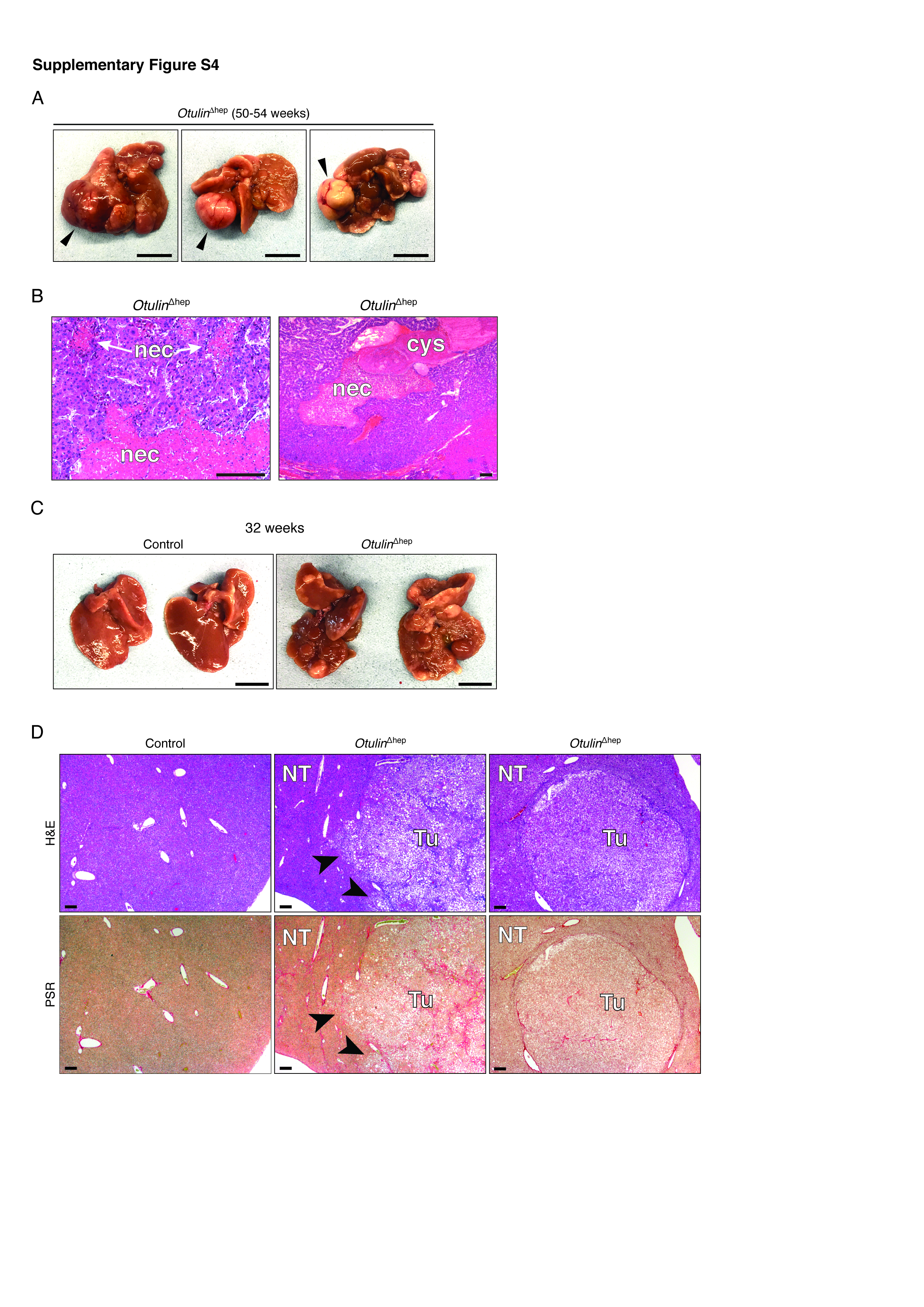

Supplement: Supplementary file 4 — S4 [file 41418_2020_532_MOESM4_ESM.tif]

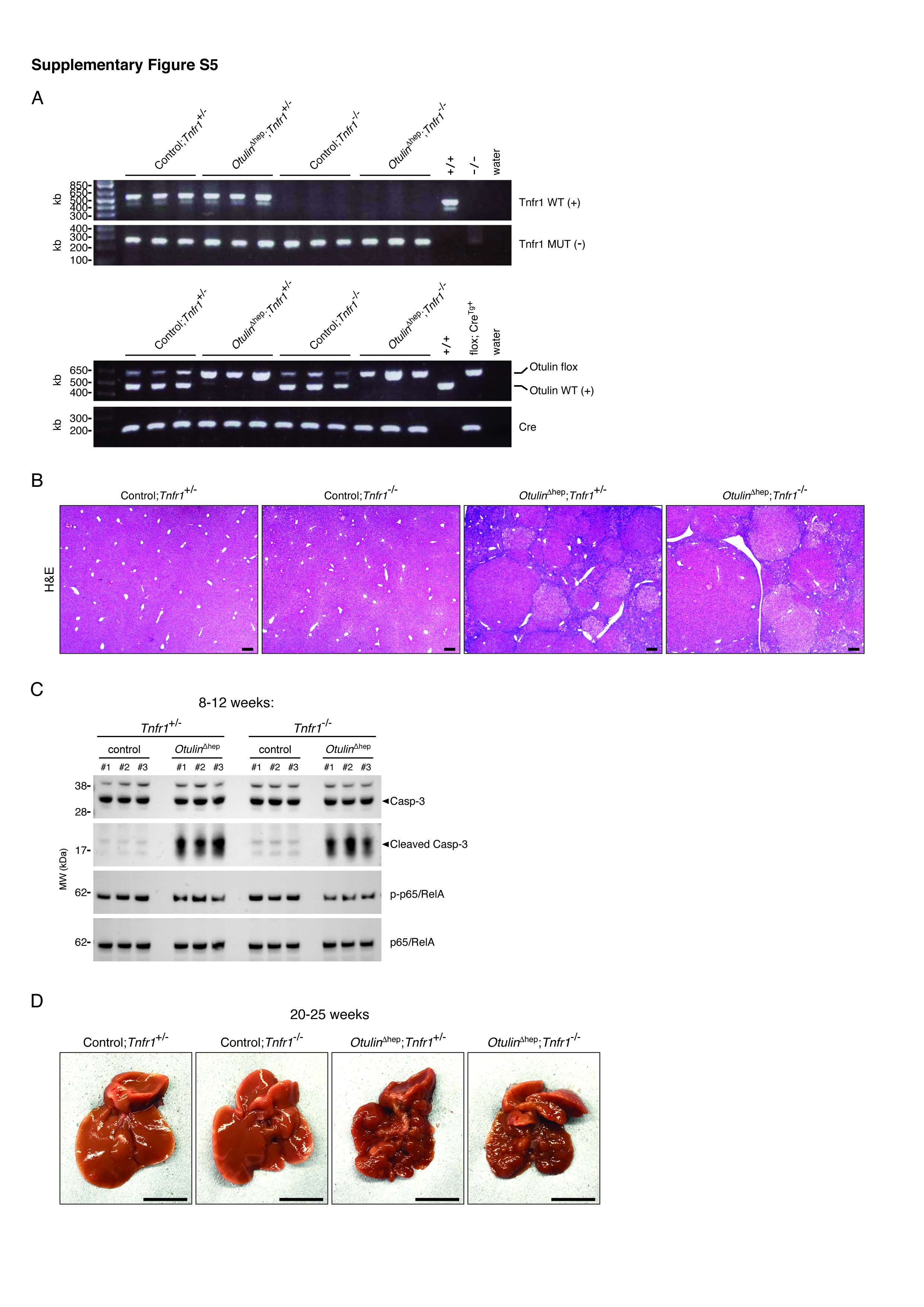

Supplement: Supplementary file 5 — S5 [file 41418_2020_532_MOESM5_ESM.tif]

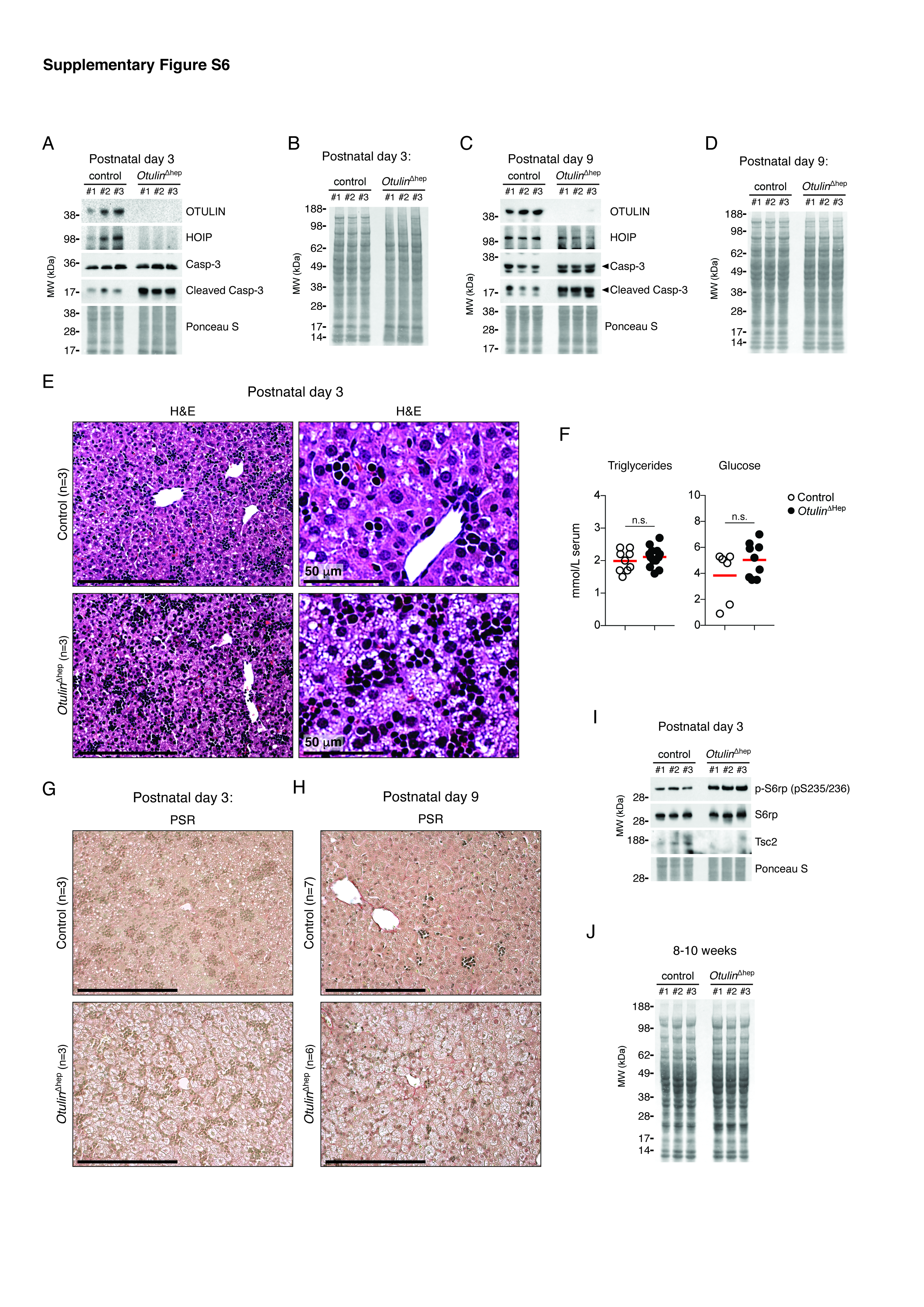

Supplement: Supplementary file 6 — S6 [file 41418_2020_532_MOESM6_ESM.tif]

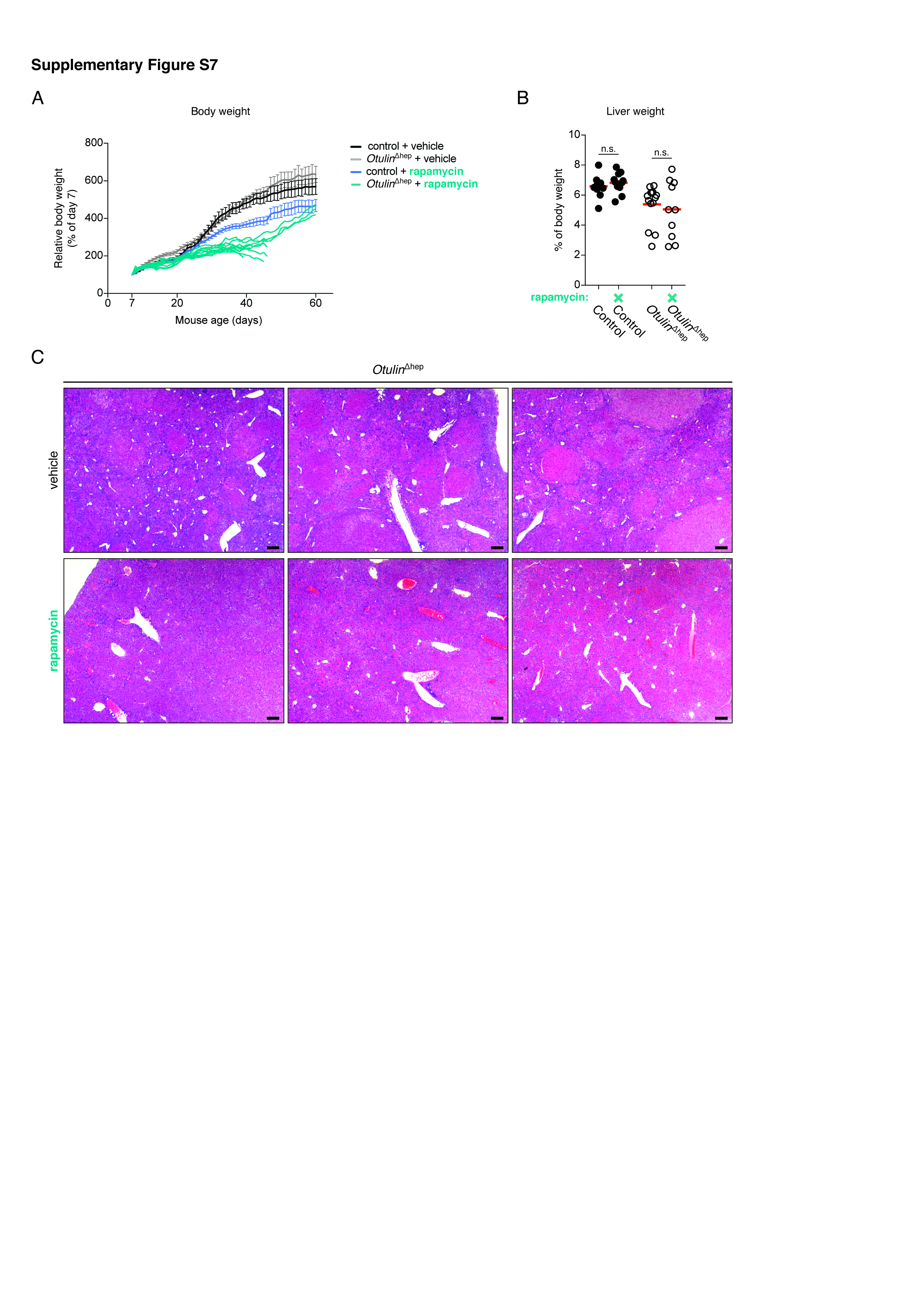

Supplement: Supplementary file 7 — S7 [file 41418_2020_532_MOESM7_ESM.tif]
