# Supplementary material for: OTULIN protects the liver against cell death, inflammation, fibrosis, and cancer
Source: Cell Death Differ. 2020 Mar 30;27(5):1457–74. doi: 10.1038/s41418-020-0532-1 (PMC7206033; doi:10.1038/s41418-020-0532-1)
Supplement: Supplementary file 8 — Supplemental Material [file 41418_2020_532_MOESM8_ESM.pdf]

OTULIN protects the liver against cell death, inflammation, fibrosis, and cancer

Rune Busk Damgaard[1,*], Helen E. Jolin[1], Michael E.D. Allison[2], Susan E. Davies[3], Hannah L. Titheradge, Andrew N.J. McKenzie[1,6], David Komander[1,4,5,6,7,*]

SUPPLEMENTARY INFORMATION

Supplementary Figures S1-7
Supplementary Figure Legends
Supplementary Table S1 (antibodies)
Supplementary Table S2 (primer sequences)

Supplemental Information: Legends

Figure S1. Analysis of Control^{Chim} and *Otulin*-KO^{Chim} mice. Related to Figure 1.

(A) Micrographs of H&E stained tissue sections from Control^{Chim} and *Otulin*-KO^{Chim} mice at the end of the experiment shown in Figure 1B. Micrographs are representative of two mice in each group.

(B) Uncropped Ponceau S stained membrane from Figure 1G.

(C) Percentage of parental (CD45.2+) cells in the spleens of Control^{Chim} and *Otulin*-KO^{Chim} at the end of the experiment shown in Figure 1B.

(D) Representative dot plot from flow cytometric analysis of parental (CD45.2+) and B6.SLJ (CD45.1+) splenocytes used to generate the plot in (C).

Figure S2. Generation and analysis of mice with hepatocyte-specific deletion of *Otulin* (*Otulin*$^{\Delta hep}$ mice). Related to Figure 2.

(A) Schematic showing the strategy used to generate mice with hepatocyte-specific deletion of *Otulin*. Numbers denote *Otulin* exons.

(B) Uncropped Ponceau S stained membrane from Figure 2B.

(C) Uncropped Ponceau S stained membrane from Figure 2C.

(D) Immunohistochemical analysis of OTULIN expression in *Otulin*$^{\Delta hep}$ and control mice at the age of 8-10 weeks shows clonal areas of hepatocytes that retain OTULIN protein expression (asterisk), likely due to incomplete penetrance and recombination efficiency of the *Alb*-Cre transgene. Micrographs are representative of two mice of each genotype.

(E) Micrographs of H&E stained liver sections from *Otulin*$^{\Delta hep}$ and control mice at the age of 8-10 weeks show histological features present in the diseased livers of *Otulin*$^{\Delta hep}$ mice. Black arrowhead indicates inflammatory focus. Blue arrowheads indicate Mallory-Denk bodies. LCC, large cell change. Micrographs are representative of six mice of each genotype.

(F) Micrographs of H&E stained liver sections from *Otulin*$^{\Delta hep}$ and control mice at the age of 8-10 weeks show formation of pre-malignant tumours (dysplastic nodules) in *Otulin*$^{\Delta hep}$ livers. Micrographs are representative of six mice of each genotype. Tu, tumour. NT, non-tumour.

(G) High magnification micrographs of PSR stained liver sections (shown in Figure 2E) from *Otulin*$^{\Delta hep}$ and control mice aged 8-10 weeks showing pericellular collagen deposition.

(H) Relative liver weights from *Otulin*$^{\Delta hep}$ (n=6) and control mice (n=6) at the age of 8-10 weeks. Each data point represents one mouse. Red bars indicate means. Data were analysed using the unpaired, two-sided Student's *t* test. n.s., non-significant.

(I) Quantification of nuclear diameter of hepatocytes from *Otulin*$^{\Delta hep}$ and control mice at the age of 8-10 weeks. Two fields of view were quantified from each of six *Otulin*$^{\Delta hep}$ mice and six control mice. Each data point represents one mouse. Red bars indicate means. Data were analysed using an unpaired, two-sided Student's *t* test. n.s., non-significant.

(J) Representative staggered histograms from flow cytometric analysis of DNA content in nuclei isolated from *Otulin*$^{\Delta hep}$ and control mice at the age of 8-10 weeks showing increased proportions of nuclei with DNA content ≥8n.

(K) Quantification of flow cytometric analysis as shown in (I). Data represent mean +SEM. Data were analysed using the unpaired, two-sided Student's *t* test.

(L) Gating strategy on a representative liver sample for flow cytometric analysis as shown in (I-J).

Figure S3. Analysis of liver disease in *Otulin*$^{\Delta hep}$ mice. Related to Figure 3.

(A) Uncropped Ponceau S stained membrane from Figure 3F.

(B) Uncropped Ponceau S stained membrane from Figure 3H.

Figure S4. Analysis of hepatocellular carcinoma in *Otulin*$^{\Delta hep}$ mice. Related to Figure 4.

(A) Representative macroscopic appearance of *Otulin*$^{\Delta hep}$ livers at the age of 50-54 weeks. Arrowheads indicate highly vascularised tumours. Scale bars indicate 1 cm.

(B) Micrographs of H&E stained tumours from *Otulin*$^{\Delta hep}$ mice aged 50-54 weeks. nec, necrotic area. cys, cystic lesion.

(C) Representative macroscopic appearance of *Otulin*$^{\Delta hep}$ and control livers at the age of 32 weeks. Scale bars indicate 1 cm.

(D) Micrographs of H&E (top panels) and PSR (bottom panels) stained liver sections from *Otulin*$^{\Delta hep}$ (n=5) and control mice (n=8) at the age of 32 weeks. Arrowheads indicate areas of poor tumour demarcation. Tu, tumour. NT, non-tumour.

Figure S5. Analysis of OTULIN and TNFR1 double-deficient livers. Related to Figure 5.

(A) PCR genotyping of *Otulin*$^{\Delta hep}$ mice, *Otulin*$^{\Delta hep}$;*Tnfr1*$^{-/-}$, and their respective controls show co-deletion of *Otulin* and *Tnfr1* as expected. kb, kilobases.

(B) Micrographs of H&E stained liver sections from *Otulin*$^{\Delta hep}$ mice, *Otulin*$^{\Delta hep}$;*Tnfr1*$^{-/-}$ mice, and their respective controls at the age of 8-12 weeks showing no difference in histopathological changes between *Otulin*$^{\Delta hep}$ mice and *Otulin*$^{\Delta hep}$;*Tnfr1*$^{-/-}$ mice.

(**C**) Immunoblot analysis of caspase-3 cleavage and NF-κB (p65) activation in whole-liver lysates from *Otulin*$^{\Delta hep}$ mice, *Otulin*$^{\Delta hep}$;*Tnfr1*$^{-/-}$, and their respective controls (n=3 in each group) at the age of 8-12 weeks.

(D) Representative macroscopic appearance of livers from *Otulin*$^{\Delta hep}$ mice, *Otulin*$^{\Delta hep}$;*Tnfr1*$^{-/-}$ mice, and their respective controls at the age of 20-25 weeks. Scale bar indicates 1 cm.

Figure S6. Analysis of neonatal *Otulin*$^{\Delta hep}$ and control mice. Related to Figure 6.

(A) Immunoblot analysis of OTULIN, HOIP, and caspase-3 in whole-liver lysates from three *Otulin*$^{\Delta hep}$ and three control mice aged 3 days.

(B) Uncropped Ponceau S stained membrane from Figure S6A.

(C) Immunoblot analysis of OTULIN, HOIP, and caspase-3 in whole-liver lysates from three *Otulin*$^{\Delta hep}$ and three control mice aged 9 days.

(D) Uncropped Ponceau S stained membrane from Figures S6C and 6H.

(E) Micrographs of H&E stained liver sections from *Otulin*$^{\Delta hep}$ and control mice at the age of 3 days (P3).

(F) Analysis of triglyceride and glucose levels in serum from terminal bleeds of *Otulin*$^{\Delta hep}$ (n=9) and control (n=6) mice at the age of 9 days.

(G-H) Micrographs of PSR stained liver sections from *Otulin*$^{\Delta hep}$ and control mice at the age of 3 days (P3) (F) and 9 days (P9) (G).

(I) Immunoblot analysis of mTOR pathway components and activation in whole-liver lysate from three *Otulin*$^{\Delta hep}$ mice and three controls aged 3 days.

(J) Uncropped Ponceau S stained membrane from Figure 6I.

Figure S7. Analysis of rapamycin-treated *Otulin*$^{\Delta hep}$ and control mice. Related to Figure 7.

(A) Relative body weight for *Otulin*$^{\Delta hep}$ and control mice treated with rapamycin or vehicle as indicated. Each rapamycin-treated *Otulin*$^{\Delta hep}$ mouse is represented by an individual (cyan) line. The mean weights (±SEM) are shown for the other experimental groups. Data were pooled from two independent experiments.

(B) Relative liver weights from *Otulin*$^{\Delta hep}$ and control mice at the age of 6 weeks treated with rapamycin or vehicle as indicated. Each data point represents one mouse. Red bars indicate means. Data were analysed using the unpaired, two-sided Student's *t* test. n.s., non-significant.

(C) Representative micrographs of H&E stained liver sections from three vehicle-treated and three rapamycin-treated *Otulin*$^{\Delta hep}$ mice at the age of 6 weeks.

Antibody	Catalog #	Clone	Supplier
OTULIN	ab151117		Abcam, Cambridge, UK
mouse HOIP	N/A		Tokunaga et al., 2011
HOIL-1/RBCK1	MABC576	2E2	Merck Milipore, Burlington, MA
SHARPIN	14626-1-AP		ProteinTech, Manchester, UK
CYLD	8462	D1A10	Cell Signaling Technology, Davers, MA
IκBα	9242		Cell Signaling Technology
p65/RelA	8242	D14E12	Cell Signaling Technology
phospho-p65/RelA (S563)	3033	93H1	Cell Signaling Technology
ERK1/2	4695	137F5	Cell Signaling Technology
phospho-ERK1/2 (T202/Y204)	4370	D13.14.E4	Cell Signaling Technology
p38	ab31828	M138	Abcam
phospho-p38 (T180/Y182)	ab195049	ERP18120	Abcam
Caspase-3	14220	D3R6Y	Cell Signaling Technology
cleaved Caspase-3 (D175)	9664	5A1E	Cell Signaling Technology
S6rp	2217	5G10	Cell Signaling Technology
phospho-S6rp (S235/S236)	4858	D57.2.2E	Cell Signaling Technology
TSC1/Hamartin	6935	D43E2	Cell Signaling Technology
TSC2/Tuberin	3990	D57A9	Cell Signaling Technology
Rheb	13879	E1G1R	Cell Signaling Technology
mTOR	2972		Cell Signaling Technology
phospho-mTOR (S2448)	2971		Cell Signaling Technology
CAD	11933		Cell Signaling Technology
Phospho-CAD (S1859)	70307	D5O6C	Cell Signaling Technology
Akt	4691	C67E7	Cell Signaling Technology
Phospho-Akt (S473)	4060	D9E	Cell Signaling Technology
Ubiquitin	NB300-130	Ubi-1	Novus Biologicals, Littleton, CO
linear ubiquitin (M1-polyUb)	MABS199	1E3	Merck Millipore
Ki67	RM-9106-R7	SP6	Thermo Scientific, Waltham, MA
anti-rabbit IgG HRP-coupled	NA934		GE Healthcare, Chicago, IL
anti-mouse IgG HRP-coupled	NXA931		GE Healthcare

Table S1. Primary and secondary antibodies. The target, catalog number, clone, and supplier for primary and secondary antibodies used in this study.

Target	Forward primer	Reverse primer
18S rRNA	5′-GTAACCCGTTGAACCCCATT-3′	5′-CCATCCAATCGGTAGTAGCG-3′
Tnf	5′-CCACCACGCTCTTCTGTCTAC-3′	5′-AGGGTCTGGGCCATAGAACT-3′
Il6	5′-TAGTCCTTCCTACCCCAATTTCC-3′	5′-TTGGTCCTTAGCCACTCCTTC-3′
Il1b	5′-CAATGGACAGAATATCAAC-3′	5′-ACAGGACAGGTATAGATT-3′
Tnfaip3 (A20)	5′-TTCCTCAGGACCAGGTCAGT-3′	5′-AAGCTCGTGGCTCTGAAAAC-3′
Cd68	5′-TGTCTGATCTTGCTAGGACCG-3′	5′-GAGAGTAACGGCCTTTTTGTGA-3′
Acta2 (Smooth muscle actin)	5′-CCCCTGAAGAGCATCGGACA-3′	5′-TGGCGGGGACATTGAAGGT-3′
Ccnd1 (Cyclin D1)	5′-GCCGAGAAGTTGTGCATCTAC-3′	5′-GGAGAGGAAGTGTTCGATGAA-3′
Ctgf	5′-GCCCTAGCTGCCTACCGACT-3′	5′-GCCCATCCCACAGGTCTTAGA-3′
Gpc3	5′-CTGAGCCGGTGGTTAGCC-3′	5′-TCACTTTCACCATCCCGTCA-3′
Igf2	5′-ACATGCTGCCCAAGTAACC-3′	5′-CTGACAAAGATGGCCCATAG-3′
Afp	5′-CTCAGCGAGGAGAAATGGTC-3′	5′-GAGTTCACAGGGCTTGCTTC-3′
H19	5′-CAGGGCTAGTCCGCTCAA-3′	5′-AACAGACGGCTTCTACGACAA-3′
Klf4	5′-CGGACCACCTTGCCTTACACA-3′	5′-TGACTTGCTGGGAACTTGACC-3′
Aldh1 (Aldh17a)	5′-GGTGAACATTGTCCCTGGTTAT-3′	5′-GACACTTTGTCGATGTCCATGT-3′
Cd133 (Prom1)	5′-TGGAGCTACCTGCGGTTTAGA-3′	5′-GGACCTGTGATTGCGATAATGA-3′

Table S2. Primer for RT-PCR sequences for RT-PCR.
